# SNOOPPI: a sequence-normalized database of on- and off-target protein–protein interactions

**DOI:** 10.1093/bioinformatics/btag736

**Published:** 2026-10-05

**Authors:** Sophia Vincoff, Pranam Chatterjee

**Affiliations:** Department of Bioengineering, University of Pennsylvania , 210 South 33rd Street, Skirkanich Hall, Philadelphia, PA 19104, United States; Department of Bioengineering, University of Pennsylvania , 210 South 33rd Street, Skirkanich Hall, Philadelphia, PA 19104, United States; Department of Computer and Information Science, University of Pennsylvania , 3330 Walnut Street, Levine Hall, Phildelphia, PA 19104, United States

## Abstract

**Motivation:**

Protein–protein interactions (PPIs) are central to biological processes and therapeutic interventions. While many databases document known PPIs, these resources often lack evidence-based negatives and clear identification of the specific proteoforms tested.

**Results:**

Here, we introduce SNOOPPI, a Sequence-Normalized database of on- and off-target protein–protein interactions, which represents the first unified dataset of binary PPIs that is isoform, post-translational modification, mutation, and binding site aware. By defining a PPI as a direct, physical interaction between two amino acid sequences, SNOOPPI overcomes several persistent limitations of existing PPI databases. SNOOPPI was curated from the IntAct database, taking full advantage of its experimental metadata and feature annotations to reclassify and uncover new PPIs. The final dataset comprises over 33.6K positive interactions and 5.3K negative interactions. SNOOPPI also retains over 835K unresolved interactions, explicitly capturing gaps in the experimental literature. Beyond its usefulness as a reference dataset for the scientific community, SNOOPPI has the potential to serve as a high-confidence foundation for sequence-based modeling, benchmarking, and generative design of novel protein and peptide interactors.

**Availability and implementation:**

The full database can be accessed and downloaded through an interactive Hugging Face page: https://huggingface.co/datasets/ChatterjeeLab/SNOOPPI. All code is open-source and maintained at https://github.com/sophievincoff/snooppi. The database and associated code will be updated periodically to incorporate new IntAct releases. The specific version of the code associated with this publication is archived at the following Zenodo repository: https://doi.org/10.5281/zenodo.22116350.

## 1 Introduction

Protein–protein interactions (PPIs) underlie fundamental biological processes in health and disease (Akbarzadeh *et al.* 2024, Greenblatt *et al.* 2024). Molecules that form or disrupt PPIs are central to biological investigation and therapeutic intervention across diseases including infectious disease, cancer, metabolic disease, and neurodegeneration (Lu *et al.* 2020, Alfaris *et al.* 2024, Chan *et al.* 2025). Machine learning has accelerated both PPI network inference (Evans *et al.* 2021, Burke *et al.* 2023, Xiong *et al.* 2025a) and therapeutic molecule design (Brixi *et al.* 2023, Bhat *et al.* 2025, Chen *et al.* 2025a, b, c, 2026, Tang *et al.* 2025a, b, Vincoff *et al.* 2026), yet progress in discovering, interpreting, and predicting PPIs continues to be fundamentally constrained by data quality (Neumann *et al.* 2022, Tsishyn *et al.* 2024).

Experimental PPI detection methods vary widely in throughput, reliability, and reported evidence. High-throughput assays such as yeast-two-hybrid and affinity purification mass spectrometry scale efficiently but exhibit elevated false-positive rates (Brückner *et al.* 2009, Gnanasekaran and Pappu 2023), whereas lower-throughput approaches such as X-ray crystallography and SPR provide higher confidence but limited coverage (Peng *et al.* 2017, Akbarzadeh *et al.* 2024). These assays produce diverse outputs, ranging from binary interaction calls to kinetic parameters and atomic structures (Akbarzadeh *et al.* 2024). Different methods also introduce distinct blind spots, including ambiguity in co-complex membership, loss of native cellular context, and limited sensitivity to weak or disordered interactions (Peng *et al.* 2017, Haubrich *et al.* 2023, Akbarzadeh *et al.* 2024, Busch *et al.* 2025).

This heterogeneity forces PPI databases to make explicit choices about representation, confidence, and scope. Some resources prioritize functional associations, including STRING (Szklarczyk *et al.* 2025), Reactome (Milacic *et al.* 2024), and KEGG (Kanehisa *et al.* 2023). Others aim to catalog physical interactions through literature curation and integration (Xenarios *et al.* 2002, Zanzoni *et al.* 2002, Breuer *et al.* 2013, Oughtred *et al.* 2021, del Toro *et al.* 2022, Samarasinghe *et al.* 2025, Kotlyar *et al.* 2026). Structural repositories such as the RCSB PDB (Berman *et al.* 2000) capture experimentally resolved complexes, requiring additional filtering and augmentation to infer biologically relevant PPIs (Liu *et al.* 2015, Bushuiev *et al.* 2024, Kovtun *et al.* 2024, Wei *et al.* 2024, Zhang *et al.* 2024, Bhat *et al.* 2025, Chen *et al.* 2026). Many datasets define a narrow scope, focusing on measurements like binding affinity (Zhu *et al.* 2026) or specific biological applications (Veres *et al.* 2015, Du *et al.* 2021). Confidence scoring schemes (Alanis-Lobato *et al.* 2017, del Toro *et al.* 2022) and community standards have helped streamline the labor-intensive upkeep and harmonization of these diverse resources. Several PPI databases, including IMEx Consortium members (Xenarios *et al.* 2002, Zanzoni *et al.* 2002, Breuer *et al.* 2013, del Toro *et al.* 2022, Samarasinghe *et al.* 2025, Kotlyar *et al.* 2026) and BioGRID (Oughtred *et al.* 2021), adopt the PSI-MI XML standard for representation and exchange of molecular interaction data (Hermjakob *et al.* 2004). This data format ensures PPI records are clean, consistent, interpretable, shareable, and parsable. PSI-MI XML 3.0 extends the framework to support more complex interaction records, including molecular features, mutations, dynamic interactions, and complexes (Sivade *et al.* 2018).

Despite these efforts, core problems remain unresolved. Protein identities are inconsistently specified, negative evidence is rarely explicit, and assay failure is often indistinguishable from absence of interaction. Most PPI databases are overwhelmingly positive-only due to the scarcity of experimentally verified non-interactions, with limited exceptions derived from literature and structure-based analyses (Blohm *et al.* 2014, del Toro *et al.* 2022, Kovtun *et al.* 2024). Consequently, negative datasets are frequently constructed using heuristic criteria such as random pairing, separation by localization or function, or exclusion by homology, introducing biases that simplify prediction tasks rather than reflect biological constraints (Neumann *et al.* 2022). Structure-based non-interactions can be particularly misleading, as lack of a resolved interface does not preclude interaction *in vivo* (Wei *et al.* 2024, Zhang *et al.* 2024). Although PSI-MI provides a rich annotation framework, it remains difficult to extract sequence-specific, binary, direct interactions from resources such as IntAct without substantial computational effort (del Toro *et al.* 2022).

To address all of these limitations, we introduce a comprehensively Sequence-Normalized database of on- and off-target PPIs, termed SNOOPPI. SNOOPPI is motivated by the observation that proteins are ultimately defined by their exact amino acid sequences, including isoforms, mutations, and post-translational modifications (PTMs), yet this information is often abstracted away in existing PPI resources. Evidence for interaction loss, arising from targeted mutations, PTM perturbations, or binding-site deletions, is frequently embedded within detailed annotations but not surfaced as explicit negative evidence. As such, we present a systematic reorganization of IntAct into a PPI and PepPI database indexed by the precise sequences of both binding partners. SNOOPPI focuses on direct, physical, binary interactions and explicitly records the experimental evidence supporting interaction and non-interaction claims. By centering sequence identity and documenting the evidence-based decisions underlying each PPI classification, SNOOPPI provides a reproducible and interpretable foundation for biological analysis and language-model-based prediction of protein interactions.

## 2 Methods

### 2.1 Source data

All source data were derived from IntAct (del Toro *et al.* 2022) Release 251 (September 2025). IntAct was selected because it uniquely combines controlled vocabularies [PSI-MI 3.0 (Sivade *et al.* 2018)], explicit amino acid sequences for most interactions, isoform, PTM, mutation, and binding-region awareness, inclusion of PPIs involving intrinsically disordered or structurally intractable proteins, literature-derived non-interacting pairs, clear distinction between directly detected binary interactions and those inferred via matrix or spoke expansion, comprehensive molecular interaction (MI) confidence scores, integration with other PPI databases, and frequent updates. Together, these properties are required for constructing a reliable, sequence-resolved PPI dataset. Several non-IMEx resources were considered (Peri *et al.* 2004, Oughtred *et al.* 2021, Szklarczyk *et al.* 2025, Zhu *et al.* 2026), but each lacked one or more of these criteria.

Although IntAct provides a user-friendly web interface and complete downloads, its data are fragmented across thousands of files with distinct formats (including XML, MITAB, TXT, and TSV), making it difficult to obtain a unified, sequence-based PPI dataset. Amino acid sequences are stored only in raw data files, while interaction expansion is indicated in the processed files. Meanwhile, the small number of reported negative PPIs (894 in intact-micluster_negative.txt) contrasts sharply with the large number of feature annotations, including 2 05 660 binding regions, 10 286 PTMs, and 83 644 mutations. Mutated and post-translationally modified sequences are not provided directly by IntAct, with sequence alterations instead being described in the feature annotation files. Several of these entries are marked as disrupting PPI formation. This suggests that many experimentally supported non-interactions are implicitly encoded but not surfaced.

To resolve these issues, we reprocessed the raw data files corresponding to individual PPI detection experiments and harmonized them with other IntAct data formats, hereby referred to as Processed, Pairs, and Features files (Fig. 1a). This pipeline yielded 33 625 positive and 5378 negative PPIs that are direct, binary interactions with known sequences for both partners. Interactions with missing sequences, conflicting or insufficient evidence, or interaction designations weaker than MI: 0407 (direct interaction) were reassigned to SNOOPPI-Unknown, which contains 8 35 083 PPIs (Fig. 1b). Most SNOOPPI interactions are protein-protein, although hundreds of peptide–protein and some peptide-peptide interactions are present as well.

**Figure 1:**
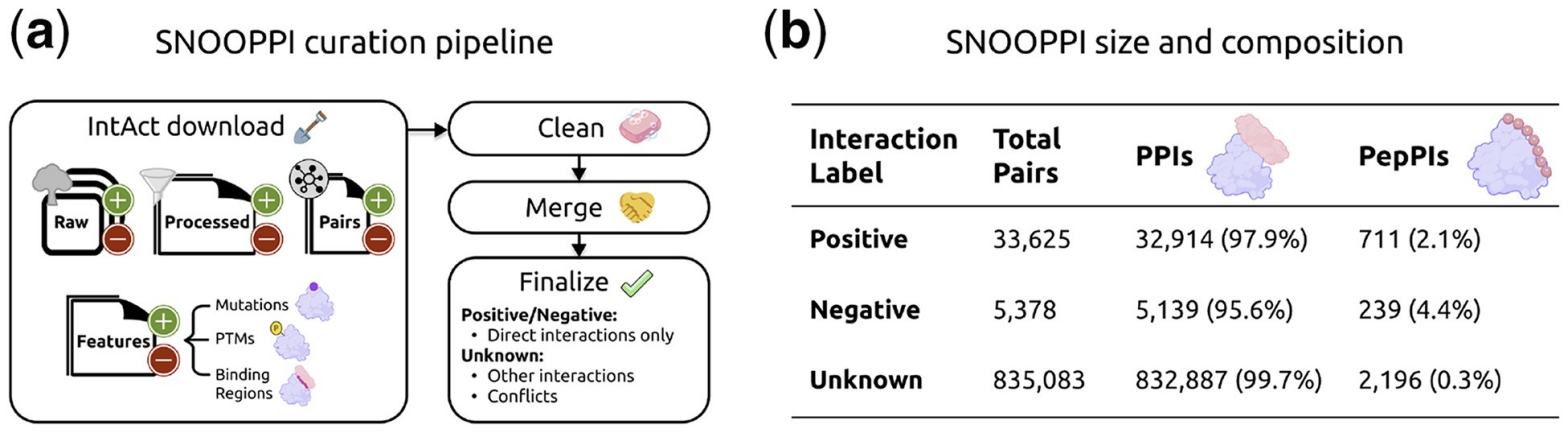
SNOOPPI database of direct, binary, sequence-based PPIs. (a) Overview of the IntAct download and reorganization process. (b) SNOOPPI database composition, including positive, negative, and unknown PPIs and PepPIs. A peptide is defined as containing at most 50 amino acids. Created in BioRender. Chatterjee, P. (2026) https://BioRender.com/u7pazpr.

### 2.2 Defining a PPI

The term “PPI” has been used to describe colocalization, functional association, complex membership, and intramolecular contacts (Akbarzadeh *et al.* 2024). Here, we define a PPI strictly as a direct, physical contact between two (poly)peptide chains in an experimental setting. A direct interaction is defined by PSI-MI term MI: 0407 as: “Interaction between molecules that are in direct contact with each other.” It is a child term of physical association (MI: 0915), which encompasses molecules “in close proximity but not necessarily in direct contact with each other.” SNOOPPI focuses on “direct interactions” because this is the most inclusive PPI category that confirms physical contact between the chains in question. Because SNOOPPI’s definition of PPI operates at the level of a physical chain rather than a gene or transcript, the exact amino acid sequence of each partner is required, including any PTMs. A protein sequence is therefore treated as text encoding the full molecular state at the time of evidence collection, such that modified and unmodified residues are represented distinctly. The identity of a PPI is defined exclusively as the order-agnostic pair of the two interacting sequences.

This binary definition permits homomeric interactions, excludes strictly intramolecular interactions, and excludes complexes containing more than two distinct amino acid sequences, while retaining binary PPIs with non-1:1 stoichiometries. Interactions may be transient or stable and may span a wide range of affinities.

We define a peptide-protein interaction (PepPI) as a binary interaction in which one partner is a peptide containing at most 50 amino acids. Although no length threshold precisely separates peptides from proteins, this cutoff is a common convention in peptide science (Sharma and Sharma 2025, Liu *et al.* 2025b,Forbes Kaprive and Merheb 2026; ). It is also biologically and practically motivated: longer chains are increasingly likely to exhibit the molecular features of proteins (Alberts *et al.* 2002, Liu *et al.* 2025b), and chains beyond approximately 40–60 residues are generally more difficult to synthesize chemically (Nilsson *et al.* 2005, Hamley 2020). Adopting a 50-amino-acid peptide cutoff required reclassification of several IntAct sequences.

A “negative PPI” denotes a pair of sequences that do not form a direct, binary, physical interaction based on experimental evidence. IntAct’s raw negatives are manually curated from studies reporting lack of interaction, with most direct negatives identified via pull-down assays (PSI-MI MI: 0096). We do not filter interactions by assay type, but retain assay metadata and IntAct MI-scores, which incorporate assay-dependent evidence weighting. Most negative PPIs in SNOOPPI are newly derived from mutation, PTM, and binding-region annotations. For mutation and PTM-derived negatives, IntAct provides both the original sequence and the exact experimental modification, allowing us to reconstruct the sequence that was tested. For necessary binding regions (MI: 0429), IntAct identifies a region whose mutation or deletion abolishes interaction but does not specify the tested modification. Negatives derived from these annotations are therefore inferred sequence variants rather than the exact sequences tested experimentally.

An “unknown PPI” is a binary sequence pair present in IntAct that either lacks decisive positive or negative evidence under our definition, or has conflicting evidence supporting both labels. SNOOPPI-Unknown therefore represents evaluated but unresolved pairs, rather than untested protein combinations or a third biological interaction class. We retain these pairs to distinguish inconclusive evidence from a complete absence of interaction information. Their associated experimental metadata, publications, and PSI-MI terms allow users to examine why a pair received an unknown label and to identify candidates for further investigation. We recommend excluding SNOOPPI-Unknown from conventional supervised binary classification unless a task-specific subset is compatible with a broader interaction definition. For example, users studying physical associations at the protein-complex level, rather than strict binary interactions, may find relevant pairs in this collection. To support this use case, we include in SNOOPPI-Unknown the hypothetical label a pair would have received if MI: 0915 (physical association) were sufficient. More broadly, SNOOPPI-Unknown can support hypothesis generation and guide additional experiments.

### 2.3 Processing and filtering IntAct

We provide step-by-step documentation of the SNOOPPI curation pipeline in Appendix A and ensure full reproducibility through our open-source codebase. Figure 2 summarizes the major processing stages and their objectives.

**Figure 2:**
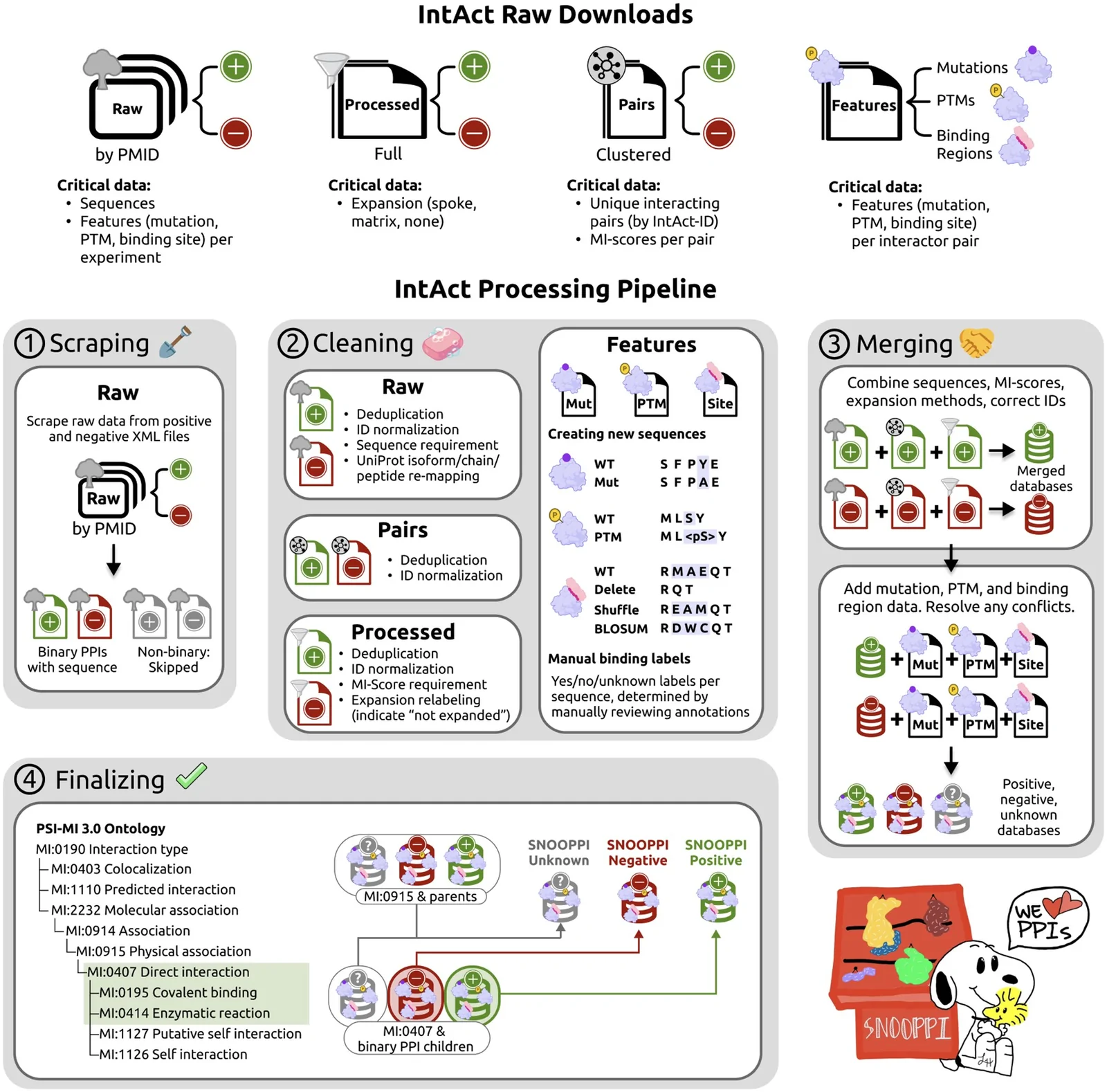
SNOOPPI full data curation pipeline. Created in BioRender. Chatterjee, P. (2026) https://BioRender.com/u7pazpr.

#### 2.3.1 IntAct raw downloads

IntAct organizes its vast data into various files, all of which are freely available. We began by downloading four collections of data, which we have termed Raw, Processed, Pairs, and Features. Raw data has been reviewed, annotated, and organized by IntAct, but remains in a format that maximizes computer-parsability rather than user-friendliness. The Processed dataset filters Raw into tables where each row represents a single piece of experimental evidence. Pairs is a clustered version of Processed, where each row corresponds to a unique PPI. Finally, Features refers to a group of three tables which describe mutations, PTMs, and binding regions respectively. Within the Raw, Processed, and Pairs datasets, positive and negative PPIs are clearly marked in separate files.

The Raw collection stores maximal experimental detail for each published article in a tree-based structure, with one file per PubMed ID. Several annotations in Raw are unavailable through the IntAct web interface or other formats. Critically, only the Raw files contain the precise amino acid sequences used in each experiment, derived from the original publication. These sequences can be mapped to specific UniProt isoforms, chains, propeptides, transit peptides, signal peptides, and other subsequences. Raw files also include per-experiment feature annotations, enabling identification of mutations, PTMs, and binding regions that affect interactions but were tested only in *n*-ary (*n *> 2) or expanded experiments.

The tabular Processed files retain much of Raw’s detail and are the only format that explicitly records whether spoke or matrix expansion was applied. Expansion status is not indicated in Raw data, and the clustered Pairs files do not distinguish interactions supported by non-expanded evidence. Pairs files define a unique interaction as the order-agnostic pairing of two IntAct identifiers. They provide a single MI-score per interaction, aggregating the quality and quantity of experimental evidence into one value which reflects the confidence of the assigned label (positive or negative).

The Feature files hold interpretable summaries of mutation, PTM, and binding-region annotations. While fully recoverable from Raw, these datapoints are resolved only at the interaction and interactor identifier level. Because multiple experiments can contribute to a single IntAct interaction, Feature files alone do not reveal which features correspond to binary, non-expanded evidence. We therefore used the Raw files to scrape feature data for relevant interactions, cross-checking against the Feature files to ensure accuracy.

#### 2.3.2 IntAct processing pipeline

Raw data were extracted via programmatic scraping (Fig. 2, part 1). Interactions involving more than two partners or any non-(poly)peptide participants were excluded (Table A1). All datasets were then cleaned (Fig. 2, part 2) through deduplication and identifier normalization. Only entries containing sequences for both partners were retained. UniProt ID Mapping was applied to obtain updated UniProt accessions, including isoform, chain, and subsequence identifiers. In the full Processed files, experiments not marked as matrix- or spoke-expanded were assigned a new explicit label, “not expanded.”

Feature processing focused on identifying full partner sequences and determining whether those sequences interact, both before and after modification. Binding outcomes were ascertained by manual review of all MI-terms and feature annotations associated with mutations, PTMs, and binding regions. Each annotation was labeled as “yes,” “no,” or “unknown.” Labels were applied at the row level (one piece of evidence) and then aggregated by amino acid sequence to assign final interaction labels.

We first processed mutations (see Table A2 for MI-terms, Table A3 for summary) and PTMs (see Table A4 for MI-terms, Table A5 for summary) using a similar workflow. Most of IntAct’s mutation and PTM entries specify the modified residues in each experiment, allowing full sequence reconstruction. Any annotations with unclear sequences were removed from consideration. Because no standardized PTM sequence encoding exists, PTMs were represented using the full PSI-MI term supplied by IntAct. For example, a tyrosine at position 1 (“Y”) could be encoded as < Y1 + MOD: 00181(O4’-sulfo-L-tyrosine)>, with angle brackets denoting a nonstandard token. To facilitate SNOOPPI’s adaptation to machine learning applications, we provide a mapping between SNOOPPI PTM tokens and those of PTM-Mamba (Peng *et al.* 2025), the only protein language model to our knowledge that explicitly incorporates PTMs into its vocabulary (Tables A6, A7).

Binding-region feature analysis required a slightly different workflow (see Table A8 for MI-terms and Table A9 for a processing summary). IntAct’s binding region annotations identify functionally necessary subsequences, but do not provide the exact sequence variants tested experimentally. We therefore retained only necessary binding regions (MI: 0429) and, following the MI: 0429 definition that deletion or mutation abolishes binding, designed disruptive sequence variants rather than discarding these annotations. We generated three predicted non-interacting variants per sequence: deletion of the binding region, random shuffling of the region, and residue-wise replacement with the least likely BLOSUM62 substitution. The shuffled control reflects common experimental practice (Wu *et al.* 2016), while the BLOSUM62-based variant (Henikoff and Henikoff 1992) minimizes chemical similarity to maximize disruption likelihood. Thus, mutation- and PTM-derived sequences in SNOOPPI correspond to experimentally tested sequence states, whereas binding-region-derived sequences are inferred negatives generated from experimentally annotated regions required for binding (Table A10). These classes are clearly distinguished in the final dataset for ease of use.

Cleaned datasets were merged (Fig. 2, part 3), and conflicts necessitated the creation of an Unknown category. Any sequence pairs with opposing experimental evidence, showing both interaction and non-interaction, were assigned to Unknown.

Finally, merged data were filtered by interaction type (Fig. 2, part 4). Only MI: 0407 (“direct interaction”) and its subtrees rooted at MI: 0195 (“covalent binding”) and MI: 0414 (“enzymatic reaction”) were accepted as evidence of direct physical interaction. MI: 1127 (“putative self interaction”) entries were reclassified as Unknown due to ambiguity between homomeric and intramolecular interactions. MI: 1126 (“self interaction”) entries were excluded entirely, as they represent confirmed unary interactions. The final SNOOPPI-Unknown dataset aggregates interactions labeled with MI: 1127, interactions under parent terms of MI: 0407, and all other interactions failing decisive classification. Only direct positive and negative interactions were retained in the final SNOOPPI-Positive and SNOOPPI-Negative datasets. IntAct’s raw data and SNOOPPI’s final composition are compared in Fig. 3a.

**Figure 3:**
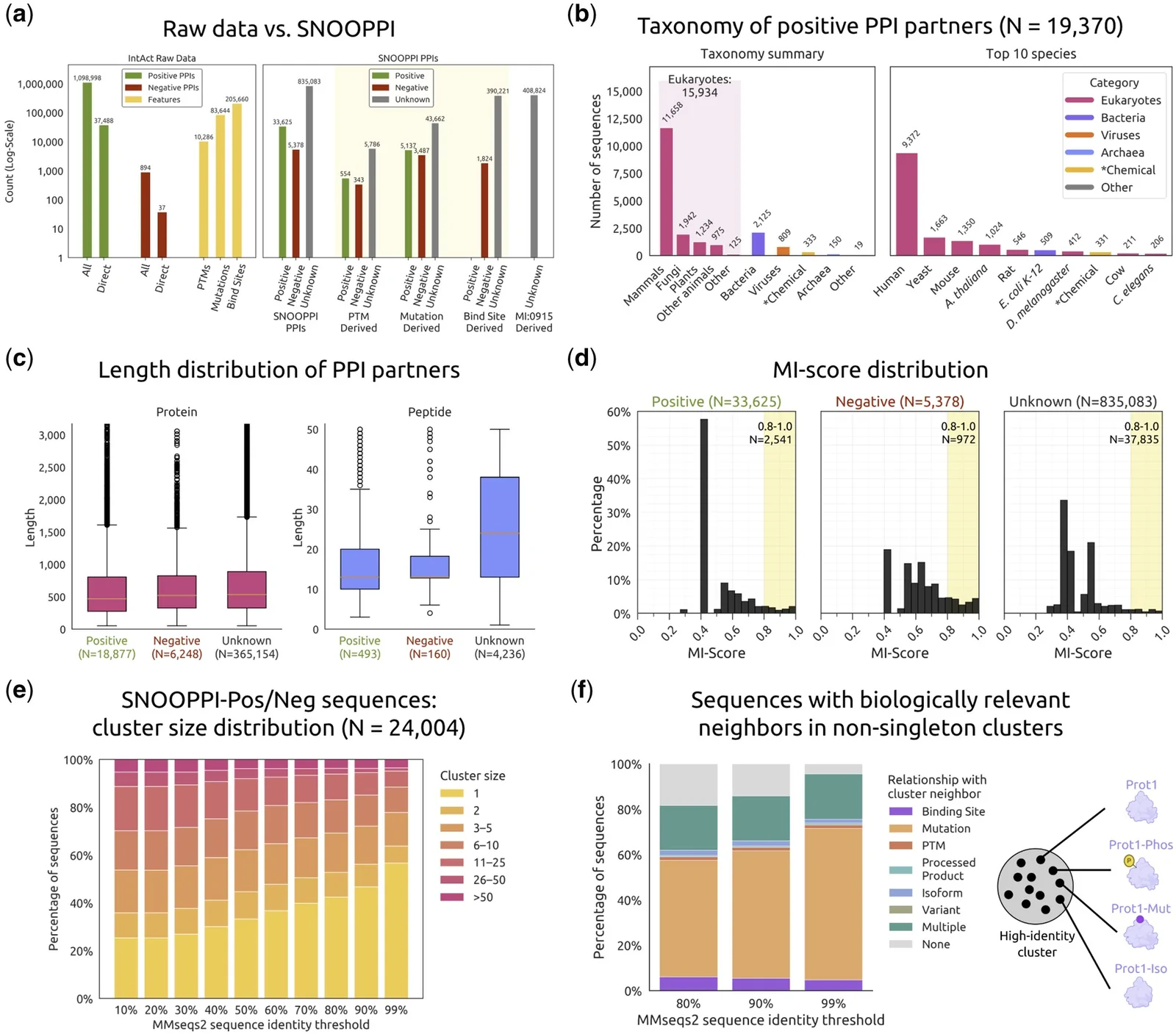
SNOOPPI data composition. (a) IntAct provides thousands of annotations that can augment its positive and negative PPI datasets (left). SNOOPPI uses PTM, mutation, and binding site annotations, as well as interaction MI-terms, to confirm or reclassify IntAct’s PPIs (right). (b) Taxonomic (left) and species (right) distributions of the individual proteins participating in SNOOPPI’s positive PPIs. *Chemical represents chemical synthesis, typically referring to a peptide. (c) Protein (left) and peptide (right) sequence lengths, up to 97th percentile, in SNOOPPI-Positive, Negative, and Unknown. (d) Distribution of MI-scores across SNOOPPI-Positive (left), Negative (middle), and Unknown (right). Total number of interactions with high MI-scores (0.8–1.0) is shown for each dataset. (e) MMseqs2 cluster size distribution at increasing homology levels for all unique sequences in SNOOPPI-Positive and SNOOPPI-Negative. (f) Sequences with biologically relevant neighbors in non-singleton clusters at high homology levels. “Multiple” indicates more than one type of biological relationship with neighbors. Example cluster (right) where proteins share PTM (Prot1 & Prot1-Phos), mutant (Prot1 & Prot1-Mut), isoform (Prot1 & Prot1-Iso), and variant (Prot1-Phos & Prot1-Mut & Prot1-Iso) relationships. Created in BioRender. Chatterjee, P. (2026) https://BioRender.com/u7pazpr.

### 2.4 SNOOPPI diversity analysis

SNOOPPI taxonomic analysis was performed using the partner species field, originally parsed from IntAct (Fig. 3b, B1). Each taxonomic ID was mapped to a species and domain using the NCBI Taxonomy (Schoch *et al.* 2020). Length (Fig. 3c) and MI-score (Fig. 3d) distributions were plotted directly from SNOOPPI fields. Sequence diversity within SNOOPPI was evaluated using MMseqs2 clustering software (Steinegger and Söding 2017) (Fig. 3e and f). All PPI and non-PPI partners within SNOOPPI with valid sequences (only containing the twenty canonical amino acids or U) were clustered at 10%, 20%, 30%, 40%, 50%, 60%, 70%, 80%, 90%, and 99% homology. To include PTM proteins, all PTM tokens were mutated to X or, in the case of multiple PTMs at the same position, a random mutation not already represented in SNOOPPI. Coverage value was set to 0.80 and coverage mode was set to 0 for all clustering runs. Results are reported for SNOOPPI-Pos/Neg (combination of SNOOPPI-Positive and SNOOPPI-Negative), the most relevant subset for machine learning training and benchmarking (Fig. 3e and f). Homology analysis including SNOOPPI-Unknown is provided in Supplementary Fig. B2. The high-homology clusters of SNOOPPI-Positive and SNOOPPI-Negative (at 80%, 90%, and 99% identity) were further analyzed for close relationships between proteins in the same cluster (Fig. 3f). For example, if two proteins that are mutated versions of each other from SNOOPPI’s curation process occur in the same cluster, they are each marked as participating in a within-cluster “Mutation” relationship. Binding site and PTM relationships work similarly. “Processed product” refers to a relationship where one protein has a canonical UniProt identifier, and another shares the same base identifier with a processed product suffix, “-PRO…” “Isoform” refers to a case where two proteins in the same cluster share the same UniProt accession, but correspond to different isoforms. “Variant” indicates cases where two proteins are related through the same wild-type protein. For example, a protein’s binding site deletion sequence and PTM sequence share a “variant” relationship.

### 2.5 Comparison with existing PPI databases

We compared SNOOPPI with five positive (PepPrCLIP (Bhat *et al.* 2025), PepMLM (Chen *et al.* 2025a), PPIKB (Zhu *et al.* 2026), PPIRef (Bushuiev *et al.* 2024), and the STRING v12.0 physical network, which we call STRING-Physical (Szklarczyk *et al.* 2023)) and three negative (Negatome 2.0 (Blohm *et al.* 2014), SCN (Chen *et al.* 2019), and TPPNI (Chatterjee *et al.* 2025)) PPI databases. Additional details about each database are provided in Supplementary Information Section A.1. The total number of unique PPIs and partners, in both SNOOPPI and comparator databases, can be found in Table B1. Cardinality is provided for the raw datasets, as well as various cleaned, filtered, and sampled subsets used for different analyses.

#### 2.5.1 Sequence overlap and taxonomic diversity analysis

For each of the eight databases, we calculated reciprocal overlap: both the percentage of SNOOPPI present in the database, and the percentage of the database present in SNOOPPI. Results are provided for individual PPI partners (Fig. 4a, Fig. B3, Table B2) and PPI sequence pairs (Fig. B4, Table B3). Taxonomic diversity analysis required the collection of NCBI Taxonomy species identifiers for each interacting protein in each dataset (Schoch *et al.* 2020). STRING-Physical species were taken from the taxonomic identifiers encoded in STRING v12 protein IDs. For all other databases, UniProt IDs were either provided directly or extracted, and then these IDs were mapped to species using the UniProt ID Mapping online tool (The UniProt Consortium 2025). PPIRef UniProt IDs were extracted from CIF files; PepPrCLIP and PepMLM UniProt IDs were extracted using the RCSB PDB’s REST API (Berman *et al.* 2000). For each database, the total unique taxonomic IDs (Fig. 4b) and the top ten species (Fig. B5) were plotted.

**Figure 4:**
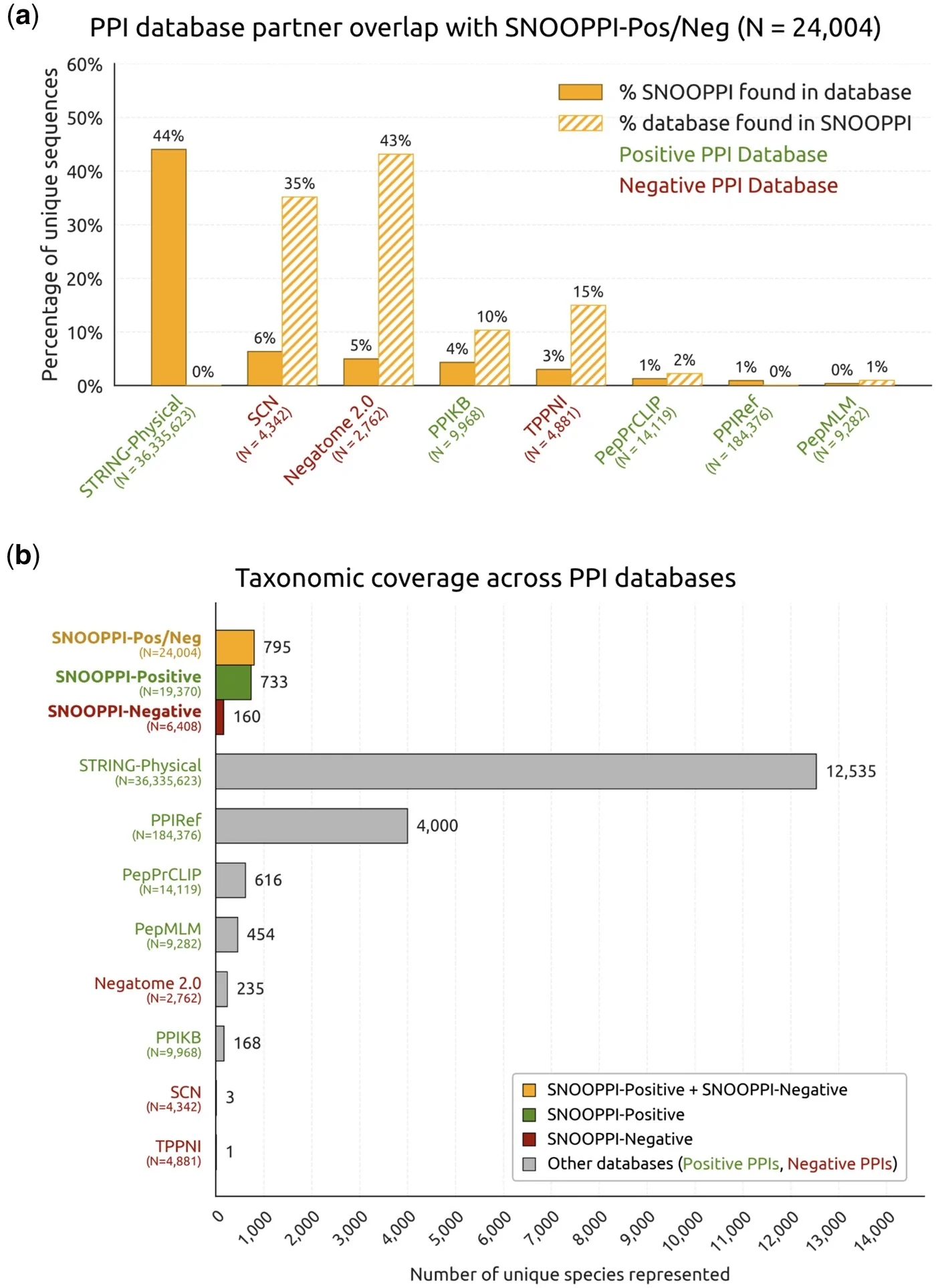
SNOOPPI sequence and taxonomic diversity in comparison to established PPI databases. SNOOPPI-Positive and SNOOPPI-Negative partners are compared with PPI/non-PPI partners from STRING-Physical, SCN, Negatome 2.0, PPIKB, TPPNI, PepPrCLIP, PPIRef, and PepMLM. Positive PPI databases are indicated in green text, and negative PPI databases in red. (a) Reciprocal overlap between SNOOPPI and existing PPI databases. (b) Number of unique species by taxonomic ID (*e.g.,* 9606 for human) per database.

**Figure 5:**
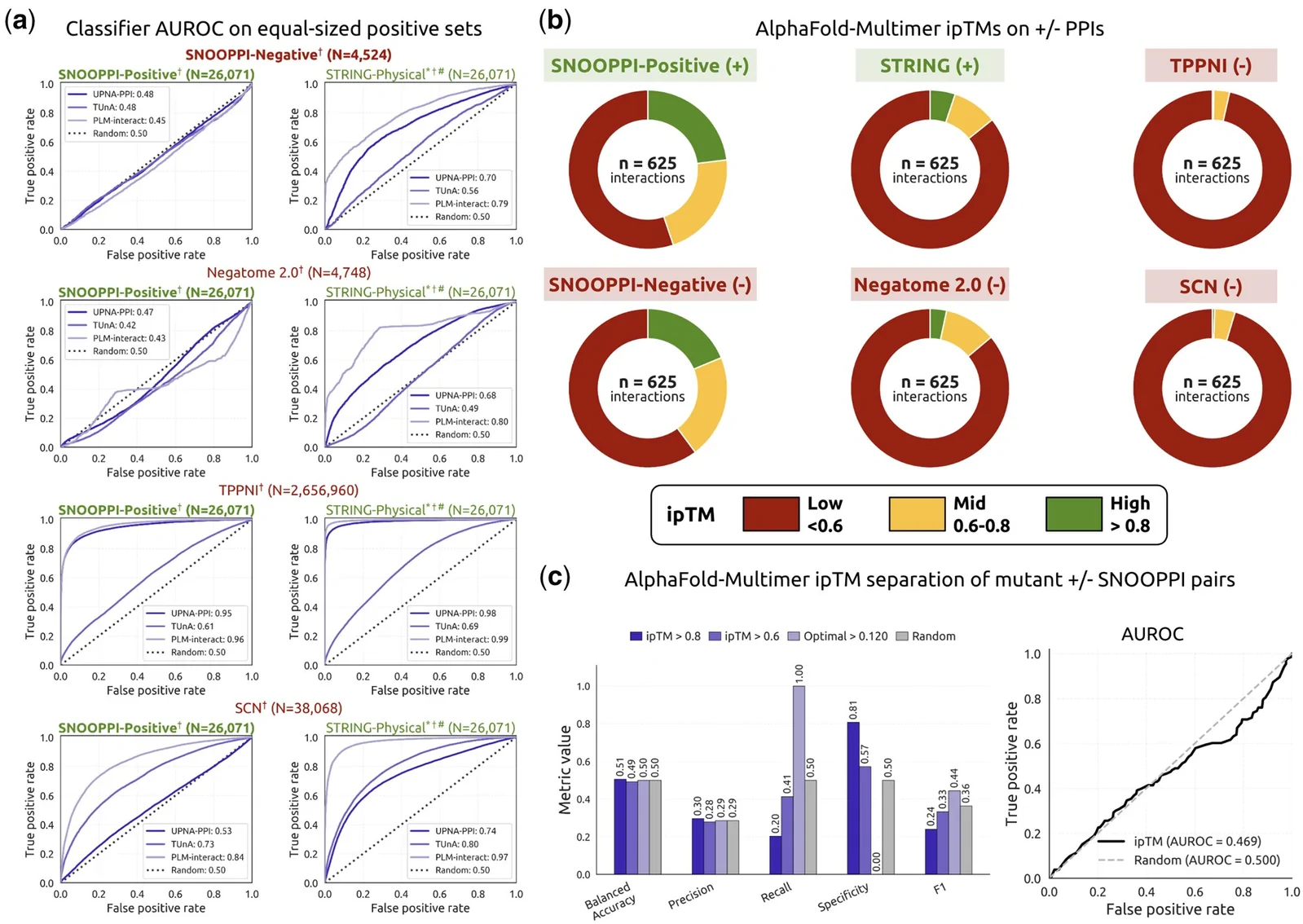
PPI predictor performance on SNOOPPI and established PPI databases. (a) AUROC curves for sequence-based models UPNA-PPI, TUnA, and PLM-interact on SNOOPPI-Negative†, Negatome 2.0†, TPPNI†, and SCN†. One curve per positive dataset (SNOOPPI-Positive†, STRING-Physical${\;}^{*†\#}$) is plotted. All datasets are subsampled for computational feasibility, and positive PPI cardinalities are matched. (b) AlphaFold-Multimer ipTMs on 625 sampled pairs, each ≤ 800AA total, for all positive and negative datasets. (c) AlphaFold-Multimer performance on 625 paired wild-type positive and mutant negative PPIs from SNOOPPI. (left) classification metrics at various ipTM thresholds, (right) AUROC curve.

### 2.6 PPI prediction with sequence and structure-based models

We conducted computational PPI prediction on five datasets: positive sets SNOOPPI-Positive and STRING-Physical (Szklarczyk *et al.* 2025), and negative sets TPPNI (Chatterjee *et al.* 2025), SCN (Chen *et al.* 2019), and Negatome 2.0 (Blohm *et al.* 2014). We selected the sequence-based binary classifiers TUnA (Ko *et al.* 2024), UPNA-PPI (Chatterjee *et al.* 2025), and PLM-interact (Liu *et al.* 2025a), as well as the structure-based folding algorithm AlphaFold-Multimer (Evans *et al.* 2021), based on their distinct approaches and high performance. Each model was run out of the box, without retraining or fine-tuning. The TUnA checkpoint used here was trained on the Bernett gold-standard dataset (Bernett *et al.* 2024), which comprises positive human PPIs from HIPPIE v2.3 (Alanis-Lobato *et al.* 2017) and randomly paired negative examples chosen to account for node-degree bias. Although HIPPIE does not list STRING as a direct source, it aggregates interactions from several databases that also contribute to STRING, potentially resulting in indirect overlap with STRING-derived datasets. UPNA-PPI was trained on experimental PPIs aggregated from several databases, including STRING, with TPPNI used as negative training examples. All PLM-interact predictions were made with the PLM-interact-650M-humanV11 model, which was trained on positive human PPIs from STRING v11 and negative examples formed by sampling protein pairs without documented interactions.

Due to computational limitations and size mismatches, datasets were subsampled for classifier prediction (see Supplementary Information Section B for more details). The subsets used in this analysis are designated SNOOPPI-Positive^†^, STRING-Physical${\;}^{*†\#}$, SNOOPPI-Negative^†^, TPPNI^†^, Negatome 2.0^†^, and SCN^†^, where ^†^ indicates PPIs with at most 1997 amino acids combined, * indicates the high-confidence set of STRING-Physical (confidence scores ≥900), and ${\;}^{\#}$ indicates a randomly sampled subset. Full output distributions are provided in Fig. B6, with each model’s performance on biologically relevant classes (e.g. mutant-derived PPIs) displayed in Fig. B7. AUROC (Fig. 5a) and AUPRC (Fig. B8) curves were plotted for each pairing of positive and negative datasets. Baselines represent a model that randomly guesses. For AUROC, a random model will always score 0.5; for AUPRC, random performance is equal to the proportion of positive samples. In Fig. 5a, positive databases have fixed cardinality while negative database sizes are allowed to vary. A supplemental analysis (Fig. B9) standardizes negative PPI database size as well.

In the AlphaFold-Multimer evaluation, we included a total of 625 sequence pairs per database, each with a combined length ≤800 amino acids (Fig. 5b). We downloaded all qualifying complexes previously folded by Qi *et al.* (2026) and randomly selected the remaining pairs to fold locally using LocalColabFold (Mirdita *et al.* 2022). Within SNOOPPI, we selected 625 mutant non-interacting pairs from SNOOPPI-Negative and their corresponding SNOOPPI-Positive pairs, allowing an additional analysis of AlphaFold’s ability to detect mutants that disrupt interaction (Fig. 5c). The fractions of each database that were predicted in the high-confidence (ipTM > 0.8), medium-confidence/uncertain (0.6 ≤ ipTM ≤ 0.8), and low-confidence (ipTM < 0.6) ranges were plotted (Fig. 5b), as well as AUROC (Fig. 5c, right) and AUPRC (Fig. B10) curves.

## 3 Results

### 3.1 SNOOPPI is a sequence-indexed interactome with biologically relevant negatives

SNOOPPI is a sequence-indexed subset of IntAct. IntAct contains 10 98 998 positive PPIs, of which 37 488 are direct, and 894 negative PPIs, of which 37 are direct, with each PPI represented as a row in the clustered Pairs files (del Toro *et al.* 2022). IntAct further provides 10 286 PTM, 83 644 mutation, and 2 05 660 binding-region annotations (Fig. 3a, left). Using these annotations and interaction-type MI-terms, SNOOPPI labels 8 35 083 interactions as unknown (Fig. 3a, right) and identifies 33 625 positive and 5378 negative PPIs that are direct, binary, non-expanded, and sequence-resolved.

PTM annotations were responsible for 554 positive, 343 negative, and 5786 unknown PPIs. Mutation annotations substantially expanded the dataset, providing 3487 high-confidence negatives, 5137 positives, and 43 662 unknowns. Binding-region features added 1824 negative and 3 90 221 unknown PPIs. Since many binding-region MI-terms do not establish necessity (Table A8), the corresponding mutants were assigned to SNOOPPI-Unknown. Finally, the MI: 0915 (“physical association”) term alone reclassified 4 08 824 interactions as unknown (Fig. 3a, right).

SNOOPPI-Positive spans 733 species across all three domains of life (Fig. 3b). Eukaryotes dominate, comprising 15 934 sequences (82%), with mammals contributing the largest share alongside hundreds of fungal, plant, and other animal proteins. Positive PPI partners also include Bacteria (2125) and Archaea (150), as well as viruses (809). An additional 333 peptides were chemically synthesized (Fig. 3b, left). The ten most represented species or sources, in descending order, are human, yeast, mouse, *A. thaliana*, rat, *E. coli*, *D. melanogaster*, chemical synthesis, cow, and *C. elegans*. Corresponding analyses for SNOOPPI-Negative and SNOOPPI-Unknown are shown in Fig. B1.

Protein and peptide lengths span a broad range (Fig. 3c). Values are shown up to the 97th percentile, with outliers extending to over 35 000 amino acids. Across databases, median protein length is approximately 500 amino acids, with an interquartile range of roughly 270–890 amino acids. Peptides in the positive and negative sets are slightly shorter (median 13 amino acids) than those in the Unknown set (median 24 amino acids) (Fig. 3c, right).

MI-scores, which summarize interaction confidence based on evidence quantity and quality, are skewed toward intermediate values across all datasets, with highest density between 0.4 and 0.6 (Fig. 3d). High-confidence interactions with MI-scores between 0.8 and 1.0 include 2541 positives (7.6%) and 972 negatives (18.1%), defining the most confident subset of SNOOPPI (Fig. 3d). The majority of SNOOPPI-Negative is derived from feature annotations, meaning that one partner has a modification that disrupts a known positive PPI. In these cases, MI-score represents confidence in the original, positive PPI. For the remaining SNOOPPI-Negative entries, which were verified from IntAct’s original negative set, MI-score represents confidence that the given interaction does not occur.

SNOOPPI exhibits considerable sequence diversity, with most SNOOPPI-Positive and SNOOPPI-Negative partners falling into small or singleton clusters across both low and high sequence identity thresholds (Fig. 3e). At 80%, 90%, and 99% identity, the relatively high number of sequences in non-singleton clusters is largely explained by the biologically relevant negatives recovered during SNOOPPI curation (Fig. 3f). More than 80% of sequences at 90% identity and more than 95% at 99% identity are meaningfully related to at least one cluster neighbor. For example, many clusters contain sequences that differ only by a phosphorylation site, binding-region rearrangement or deletion, or mutation. Although high sequence identity is often treated as redundancy in protein datasets, SNOOPPI’s highly homologous sequences encode biologically important variant relationships in which a single amino acid change can determine whether binding occurs.

### 3.2 SNOOPPI diversity relative to established PPI databases

To contextualize SNOOPPI relative to existing PPI resources, we first analyzed sequence overlap and taxonomic diversity. We selected a panel of established positive databases—STRING’s physical network (STRING-Physical) (Szklarczyk *et al.* 2025), PPIKB (Zhu *et al.* 2026), PepPrCLIP (Bhat *et al.* 2025), PPIRef (Bushuiev *et al.* 2024), and PepMLM (Chen *et al.* 2025a)—and negative databases—SCN (Chen *et al.* 2019), Negatome 2.0 (Blohm *et al.* 2014), and TPPNI (Chatterjee *et al.* 2025)—for comparison. SNOOPPI shares sequences with each resource, covering 43% of Negatome 2.0, 35% of SCN, and 15% of TPPNI. STRING-Physical, comprising 3 63 35 623 unique sequences, showed the greatest overlap with SNOOPPI, covering 44% of its sequences. Thus, at least 56% of SNOOPPI is not represented in any of the individual databases surveyed here. The lowest overlap was observed with the RCSB PDB-derived resources PPIKB, PepPrCLIP, PPIRef, and PepMLM. Full sequence membership and overlap counts are provided in Table B2.

We next assessed taxonomic diversity by counting the number of unique species represented in each database (Fig. 4b). The combined SNOOPPI positive and negative subsets (SNOOPPI-Pos/Neg) ranked third, spanning 795 species. STRING-Physical and PPIRef were the most taxonomically diverse resources, representing 12 535 and 4000 species, respectively.

### 3.3 SNOOPPI PPI prediction with sequence- and structure-based models

PPI prediction on SNOOPPI presents a unique challenge because PTM-, mutation-, and binding region-derived negatives often have high sequence similarity to their wild-type positive counterparts. To determine whether SNOOPPI remains trivially separable despite this feature, we calculated interaction probabilities on filtered subsets of each database (Table B1) using three sequence-based models: UPNA-PPI (Chatterjee *et al.* 2025), a topology-informed multi-layer perceptron (MLP), and the Transformer-based models TUnA (Ko *et al.* 2024) and PLM-interact (Liu *et al.* 2025a). These results should be interpreted as measures of out-of-the-box separability by pretrained PPI predictors, rather than the training utility of any individual database. All three models performed near chance when distinguishing SNOOPPI-Positive^†^ from SNOOPPI-Negative^†^ pairs (Fig. 5a, B8). Replacing SNOOPPI-Positive^†^ with STRING-Physical${\;}^{*†\#}$ consistently improved performance, with PLM-interact achieving the highest AUROC of 0.79. This improvement may partly reflect the inclusion of STRING or STRING-derived data, to varying extents, in the models’ training datasets (Ko *et al.* 2024, Chatterjee *et al.* 2025, Liu *et al.* 2025a). Negatome 2.0^†^ was likewise difficult to distinguish from SNOOPPI-Positive^†^, although performance improved when STRING-Physical${\;}^{*†\#}$ was used as the positive set. By contrast, TPPNI^†^ was readily separable across two out of three models, with AUROCs ranging from 0.95 for UPNA-PPI (which was trained on TPPNI negatives) to 0.99 for PLM-interact, largely irrespective of the positive dataset. PLM-interact also performed strongly on SCN^†^ paired with STRING-Physical${\;}^{*†\#}$, achieving an AUROC of 0.97 (Fig. 5a).

We next evaluated the predictive performance of AlphaFold-Multimer (Fig. 5b and c). Across 625 sampled pairs per dataset, each with a combined sequence length of ≤800 amino acids, ipTM scores, which reflect confidence in the predicted interface, were predominantly low. This pattern was observed across both positive and negative datasets. Although SNOOPPI exhibited a more balanced ipTM distribution than the comparison datasets, its positive and negative partitions were nearly indistinguishable (Fig. 5b). Consistent with this overlap, AlphaFold-Multimer performed near random across classification metrics on mutation-associated SNOOPPI pairs, yielding an AUROC of 0.47 (Fig. 5c).

## 4 Discussion

SNOOPPI is the first sequence-normalized dataset of experimentally verified PPIs and PepPIs, spanning positive, negative, and investigated but unresolved interactions. Although several PPI databases provide amino acid sequences (del Toro *et al.* 2022, [Bibr btag736-B75][Bibr btag736-B19][Bibr btag736-B76]), these resources are typically organized around publications, experiments, or protein identifiers (Berman *et al.* 2000, Brown *et al.* 2015, The UniProt Consortium 2025) rather than indexed by the exact sequences that were assayed, making it difficult to determine whether two specific sequences physically interact. PTMs, mutations, and isoform differences are often treated as annotations rather than first-class entities, obscuring their functional effects. SNOOPPI adopts a sequence-first representation, defining each interaction as a binary, direct physical contact between two precisely defined sequences, with mutations and PTMs incorporated directly. This reduces ambiguity, resolves interactions beyond the gene level, and yields a level of granularity that is difficult to obtain elsewhere in a unified, streamlined resource.

A central motivation for SNOOPPI is its utility for machine learning and sequence-based design. Many deep learning models for PPI prediction (Singh *et al.* 2022, Ko *et al.* 2024), PepPI prediction (Abdin *et al.* 2022, Xiong *et al.* 2025b), and interface identification (Abdin *et al.* 2022, Wang *et al.* 2025, Xiong *et al.* 2025b) are limited by training data assembled indirectly from identifiers, structures, or heterogeneous assays, with negative datasets often encoding strong and unexamined assumptions (Neumann *et al.* 2022). SNOOPPI provides a consistent starting point for such models, while remaining flexible to user-defined filtering and splitting. We find that four state-of-the-art models (Evans *et al.* 2021, Ko *et al.* 2024, Chatterjee *et al.* 2025, Liu *et al.* 2025a) struggle to distinguish SNOOPPI-Positive from SNOOPPI-Negative (Fig. 5). As most SNOOPPI negatives arise from precise interaction-disrupting perturbations, current predictors may lack sensitivity to consequential single- or few-residue changes. This limitation is consistent with prior evaluations of AlphaFold (Pak *et al.* 2023) and protein language models (Notin *et al.* 2023) on mutation-effect prediction. SNOOPPI therefore creates an opportunity to move PPI prediction toward the fine-grained determinants of interaction specificity. However, our structure-based analysis reflects only the performance of AlphaFold-Multimer ipTM under the sampled conditions and does not comprehensively assess the model using alternative scoring metrics or multiple conformations generated from different model seeds.

Beyond prediction, SNOOPPI supports generative and optimization frameworks for designing novel interactors (Brixi *et al.* 2023, Bhat *et al.* 2025, Chen *et al.* 2025a, 2026, Vincoff *et al.* 2026), with clear paths to extension toward antibodies, nanobodies, non-canonical amino acids, and chemically modified peptides via SMILES representations (Chen *et al.* 2025b, c, Tang *et al.* 2025a, b). The dataset also has limitations: unresolved interactions outnumber confident labels, some manual curation will remain necessary as ontologies evolve, PTM vocabularies lack standardization despite recent modeling advances (Peng *et al.* 2025), and the focus on binary interactions excludes multimeric complexes and systematic affinity annotation. Mutation annotations are biased toward alanine substitutions, and rare but clinically relevant protein classes, such as fusion oncoproteins, remain underrepresented (Vincoff *et al.* 2025). Even with these constraints, SNOOPPI establishes a precise, evidence-grounded, sequence-resolved foundation on which both biological understanding and machine learning models of physical protein interactions can be built and meaningfully evaluated.

## Supplementary Material

btag736_Supplementary_Data
