# Supplementary material for: SNOOPPI: a sequence-normalized database of on- and off-target protein–protein interactions
Source: Bioinformatics. 2026 Oct 5;42(10):btag736. doi: 10.1093/bioinformatics/btag736 (PMC13679979; doi:10.1093/bioinformatics/btag736)
Supplement: Supplementary file 1 [file btag736_supplementary_data.pdf]

Supplementary Information

SNOOPPI: A Sequence-Normalized Database of On- and Off-Target Protein-Protein Interactions

Sophia Vincoff,[1] Pranam Chatterjee[1,2,†]

[1]Department of Bioengineering, University of Pennsylvania
[2]Department of Computer and Information Science, University of Pennsylvania

Correspondence: pranam@upenn.edu
Dataset: https://huggingface.co/datasets/ChatterjeeLab/SNOOPPI

A Data curation

A.1 Comparator PPI Databases

PPI Database Selection SNOOPPI was compared with eight representative PPI databases for sequence overlap and taxonomic diversity: PPIRef [1], PPIKB [2], PepPrCLIP [3], PepMLM [4], STRING v12.0 [5], subcellular compartment-based negatives (referred to as SCN) [6], Negatome 2.0 [7], and TPPNI (topological protein-protein non-interactions) [8]. PPI classifier analyses utilized filtered subsets of STRING, Negatome 2.0, TPPNI, and SCN.

STRING is primarily focused on functional associations between proteins, but it does provide a physical subnetwork, which we call STRING-Physical. Derived from experiments, databases, and text mining, this dataset features protein pairs that have been detected in complex. Importantly, STRING-Physical does not guarantee binary interaction. PepPrCLIP and PepMLM are RCSB PDB-derived [9] peptide-protein interaction databases. PepPrCLIP filters RCSB PDB complexes directly, following the methodology from PepNN [10]. We combine PepPrCLIP's "noisy" training dataset, with buried surface area $\geq 50Å^2$, and its "strict" testing dataset, with buried surface area $\geq 400Å^2$. PepMLM trains on PepNN data [10] and tests on Propedia's gold standard data [11], filtering at 80% homology using MMseqs2 [12]. PPIKB is a recent peptide-protein affinity database derived from the RCSB PDB and published literature. We consider all of its sequence pairs to be positive due to their having a measurable binding affinity. PPIRef is an RCSB PDB-derived PPI dataset with several criteria: X-ray crystallography or electron microscopy detection method, resolution of at least 3.5Å, buried surface area $\geq 500Å^2$, and at most 6Å between two proteins that are considered to be interacting.

Negatome 2.0 is a dataset of non-interacting proteins. Its "Manual" subset consists of literature-derived non-interactions, which were collected by text mining and then manually reviewed. The "PDB" dataset contains protein chains from RCSB PDB complexes which are ≥ 8Å apart. Both datasets were filtered against positives in IntAct at curation time. SCN negatives [6] are essentially rearrangements of the protein sequences from three preexisting PPI datasets. Sequences with no evidence of interaction are randomly paired together and then filtered to ensure non-overlapping subcellular localizations. Finally, TPPNI is a computationally generated negative-interaction network obtained by applying a unipartite configuration model to a PPI network assembled from several databases, including STRING, and retaining low-probability protein pairs with no length-three paths in the original network [8].

PPI Database Download PPIRef [1] sequences were downloaded programmatically using the `ppiref` Python package version 1.2.0. Negatome 2.0's manual-stringent and PDB-stringent datasets were downloaded from the public web server and combined [7]. PPIKB v2025 [2] sequences were downloaded from the Affinity Dataset (main) Excel file on the web server download page. TPPNI [8] was downloaded directly from the GitHub page of the UPNA-PPI model paper, which constructed the dataset. SCN was curated from the PIPR GitHub [6] by combining the yeast [13] and sun datasets [14] and filtering for negative interactions only. Because the sun dataset additionally contains Negatome-derived pairs, we excluded all sun negatives matching Negatome 1.0 [15] by unordered sequence pair. For both PepPrCLIP [3] and PepMLM [4], the models' training and testing data from their codebases were combined. STRING's physical links were downloaded from the public webserver file protein.physical.links.v12.0.txt.gz.

A.2 IntAct Processing

The IntAct database provides a centralized resource for literature-derived, expert-verified PPIs across a wide variety of experimental methods and organisms. IntAct aggregates all data contributed by members of the IMEx Consortium, who have agreed to follow common curation standards. Active database members as of September 2025 include: IntAct [16], DIP [17], MINT [18], MatrixDB [19], IID [20], InnateDB [21], UniProt and Swiss-Prot groups [22], and EMBL-EBI [23]. Inactive members (contributed in the past) include: databases MPact [24], BIND [25], MPIDB [26], Molecular Connections, MBInfo, HPIDB [27], and the UCL-BHF group. As a starting point for our binary PPI dataset, IntAct Release 251 - September 2025 was downloaded. The full download presents IntAct's data in several formats. We utilize (1) **Raw data**: PSI30-formatted [28] XML files organized by year and PubMed ID, (2) **Processed data**: TXT files organized by experiment, (3) **Pairs**: TXT files clustered by interacting pair, and (4) **Features**: three separate TSV files describing binding regions, mutations, and PTMs that impact PPIs.

Each file format contains largely overlapping but sometimes distinct content. This ultimately necessitated the full processing and recombination of all Raw, Processed, Pair, and Feature files. Below, we outline each file format with a description, download information, and list of unique contributions relative to other file types. All file paths are relative to https://ftp.ebi.ac.uk/pub/databases/intact/current.

1. Raw data - XML files. Found at: psi30/pmid, in subfolders named for the year in which the article was published. Unique contributions:

1. **Amino acid sequences.** The precise amino acid sequences of each protein or peptide involved in the interaction.

2. **Feature annotations (PTM, mutation, and binding site) per experiment.** In the aggregated Feature files, it is not possible to identify the exact experiment associated with the feature. This is problematic when the same study provides both binary and *n*-ary interactions involving the same pair of proteins, and a PTM or mutation was only tested in the *n*-ary experiment. We limit our curation to binary interactions and their associated features only.

2. Processed data - TXT files indexed by experiment. Found at: psimitab/intact.txt (positive PPIs) and psimitab/intact_negative.txt (negative PPIs). Unique contributions:

1. **Expansion labels per experiment.** The raw XML files do not indicate whether a binary PPI was determined via matrix expansion, spoke expansion, or no expansion. Format (3) (Pairs) aggregates expansion labels but excludes no-expansion; *e.g.*, if an interaction was determined once with no expansion and once with spoke expansion, its entry under "Expansion method(s)" would only read: psi-mi:"MI:1060"(spoke expansion). An interaction that has been verified without expansion is more likely to be a true binary PPI. Therefore, our pipeline reassigns more comprehensive labels to each pair, indicating all expansion modes including none.

3. Pairs - TXT files clustered by interacting pair. Found at: psimitab/intact-micluster.txt (positive PPIs) and psimitab/intact-micluster_negative.txt (negative PPIs). These files are consolidated versions of intact.txt and intact_negative.txt, clustered by the pair of interactors. IntAct assigns a unique ID in the format "intact:EBI-*N*" to each unique combination of amino acid sequence and primary database accession. The primary database can be any IMEx Consortium member besides IntAct, such as UniProt, Ensembl, or DIP. For example, two proteins with distinct UniProt accessions that have identical sequences will receive separate intact:EBI identifiers; two proteins with the same UniProt accession but different sequences (*e.g.,* isoforms) will receive separate intact:EBI identifiers. One unique pair of interacting proteins refers to the set of the two partners' intact:EBI IDs (in any order). IntAct clusters on these pairs in order to assign a single confidence score to each PPI. Unique contributions:

1. **Unique PPI identification**. This file confirms that each unique combination of two intact:EBI IDs represents a distinct PPI, helping to guide our aggregation of data scraped from the raw XML.

2. **One interaction score per pair**. The clustered file provides a single "mi-score" which encapsulates all experiments that support the interaction of two distinct proteins, as represented by their intact:EBI IDs.

4. **Features - TSV files**: Found at psimitab/features/ptms.tsv, psimitab/features/mutations.tsv, and psimitab/features/binding_regions.tsv. These files present PTM, mutation, and binding region data for each experiment. They do not provide unique data relative to the other files (all of these annotations can be scraped directly from raw XML) but were helpful for cross-checking to confirm the correctness of the pipeline.

In addition to these files, the controlled vocabularies at cv/intact.obo were used to categorize MI terms relating to interaction type (*e.g.,* MI:0407: direct interaction, MI:0573: mutation disrupting

interaction).

A.2.1 Scraping the raw XML

Most of the raw data stored in the XML files was extracted in our scraping process. This includes:

1. Interaction-level information:
   (a) Interaction label (*e.g.,* "direct interaction")
   (b) Interaction MI (*e.g.,* "MI:0407")
   (c) Interaction IntAct ID (*e.g.,* "intact:EBI-16189444")
   (d) Experiments (including PubMed ID, method (*e.g.,* "anti bait coip"), hosts with taxonomic ID (*e.g.,* "9606") and short/full labels (*e.g.,* "human", "Homo sapiens"))
   (e) Year
   (f) Processing method ("xml" or "pymex")

   It should be noted that the `mif` module of the `pymex` package unexpectedly failed to parse many of the XML entries. To prevent data loss, we performed all parsing with `xml.etree.ElementTree`, and thus all processing methods are "xml".

2. Interactor-level information:
   (a) Gene name (*e.g.,* "rpb1_human")
   (b) Gene symbol (*e.g.,* "POLR2A")
   (c) Molecule type ("protein" or "peptide")
   (d) Species label (*e.g.,* "human")
   (e) Species taxonomic ID (*e.g.,* "9606")
   (f) Amino acid sequence (*e.g.,* "MHGGGPP...")
   (g) Length of amino acid sequence
   (h) Chain start and end coordinates for the provided sequence (1-indexed and end position-inclusive, *e.g.,* "1-405")
   (i) All UniProt, ENSP, ENSG, ENST, RCSB PDB, IntAct, and DIP accessions
   (j) All InterPro, Reactome, and GO annotations
   (k) Name and ID of primary database (*e.g.,* "uniprotkb", "Q16637")
   (l) Host information: taxonomic ID, full label, short label, cell type (*e.g.,* "293t" for HEK293T cells), cellular compartment (*e.g.,* "nucleus"), tissue (*e.g.,* "colon")
   (m) Mutation annotations:
      i. MI (*e.g.,* "MI:0573")

ii. Name (*e.g.,* "mutation disrupting interaction")

iii. Short name (*e.g.,* "E9M5R0:p.[Asp293_Ser294delinsAsnAla]")

iv. Beginning coordinates (*e.g.,* "293,296") and ending coordinates (*e.g.,* "294,297")

v. Original sequence (*e.g.,* "DS,SE") and new sequence (*e.g.,* "NA,AQ") at the specified coordinates

Sometimes, multiple mutations were applied at once. In these cases, the beginning/ending coordinates and original/new sequences were stored in comma-separated format (*e.g.,* begin: "1,3", end: "1,3", original: "M,P", new: "A,A"). There were also many XML entries where multiple mutation experiments were reported. These were pipe-separated (*e.g.,* begin: "1,3|60,61", end: "1,3|60,61", original: "M,P|R,R", new: "A,A|G,G"). This curation strategy provided a clear distinction between mutations that were applied together and those that were not. Note that all coordinates are 1-indexed and inclusive, meaning that if the beginning and ending coordinates are both "1", then the mutation was only applied to the first amino acid in the sequence.

(n) PTM annotations:

i. MI (*e.g.,* "MI:1224")

ii. Name (*e.g.,* "increasing-ptm,observed-ptm,N6-acetyl-L-lysine")

iii. Short name (*e.g.,* "possible acetyllys-310")

iv. Beginning coordinates (*e.g.,* "310") and ending coordinates (*e.g.,* "310")

v. Original and new sequences (rarely provided)

When multiple positions had PTMs or multiple PTM features were reported in one XML block, the same protocol was followed as for mutations.

(o) Binding region annotations:

i. MI (*e.g.,* "MI:0429")

ii. Name (*e.g.,* "necessary binding region")

iii. Short name (*e.g.,* "binding region")

iv. Beginning coordinates (*e.g.,* "186") and ending coordinates (*e.g.,* "292")

Positive and negative files were processed separately. Only entries that contained exactly two protein participants were included in the output file; any *n*-ary interactions were excluded. Additionally, interactions that did not strictly involve proteins and/or peptides were excluded. All skipped interactions were saved in a separate file with columns denoting the following: file name, interaction XML ID, IntAct ID for the interaction, and the reason. The interaction XML ID was pulled out of a block formatted according to the following example:

```
<interaction id="6" imexId="IM-23272-1">
```

The XML ID (in this example, 6) was stored for the following reason: in the same file, the same two proteins could be shown to interact in different XML interaction blocks, but only some blocks may hold valid binary PPIs.

This process produced three output files:

- "intact_processed_positivePPIs.csv"
- "intact_processed_negativePPIs.csv"
- "intact_skipped_positivePPIs.csv"

No negative interactions were skipped (Table A1).

Table A1: Interactions scraped from IntAct

Type	Accept/Reject	Total	Reason
Positive	Accept	746,032	
Positive	Reject	69,681	not binary
Positive	Reject	55,022	not PPI or PepPI
Negative	Accept	969	

A.2.2 Cleaning the Raw, Processed, and Pairs files

Here, we outline each step taken to clean the scraped raw XML data, as well as the raw Processed and Pairs TXT files downloaded from IntAct. Initially, database sizes were as follows:

- intact.txt = 1,726,476
- intact-micluster.txt = 1,136,486
- intact_negative.txt = 984
- intact-micluster_negative.txt = 931
- scraped file intact_processed_positivePPIs.csv = 746,032
- scraped file intact_processed_negativePPIs.csv = 969

Cleaning the non-clustered TXT files

To clean "intact.txt" and "intact_negative.txt", the following key steps were taken:

1. Deduplication (deleted copies of identical rows)
2. Dropped any row lacking an IntAct ID for Interactor A or Interactor B
3. Dropped any rows lacking an mi-score
4. Created a designation for binary PPIs that were truly detected as binary, and not inferred by spoke or matrix expansion. In all rows where "Expansion method(s)" was missing, we created a label: "not expanded". This had a significant impact, leaving the database with 916,419 rows (53.12%) labeled as "psi-mi:"MI:1060"(spoke expansion)" and 808,646 rows (46.88%) labeled as "not expanded."

5. Exploded along the column "IntAct Interaction identifier(s)" so that each row corresponded to just one intact:EBI ID for the interaction. Only three rows from "intact.txt" and zero from "intact_negative.txt" originally had multiple IntAct interaction identifiers.

Cleaning the micluster files

To clean "intact-micluster.txt" and "intact-micluster_negative.txt", the following key steps were taken:

1. Deduplication (deleted copies of identical rows)
2. Dropped any row lacking an IntAct ID for Interactor A or Interactor B
3. Confirmed that the unique combination of intact:EBI identifiers for Interactor A and Interactor B only appears once throughout the database

Cleaning the scraped XML files

To clean "intact_processed_positivePPIs.csv" and "intact_processed_negativePPIs.csv", the following key steps were taken. We note that in these scraped files, Interactors A and B are instead referred to as Interactors 1 and 2. In the merging process, this difference in notation clarified which columns came from the scraped XML and which came from the raw IntAct TXT downloads. To minimize confusion, we will continue to use the terminology A and B in this section.

1. Deduplication (deleted copies of identical rows)
2. Dropped any row lacking an amino acid sequence for Interactor A or Interactor B
3. Checked that each row has at least one IntAct identifier for Interactors A and B.
4. Exploded along the columns containing these identifiers. The resulting database contained exactly one IntAct ID for each interactor in each row.
5. Exploded along the column containing the IntAct identifiers for the interaction as a whole. The resulting database contained exactly one IntAct ID representing the interaction in each row.
6. Prepared for merging by flipping (and effectively doubling) the database so we could later match it to the corresponding "intact-micluster(_negative)" rows even if the interactors were in the opposite order.

A.2.3 Merging the scraped Raw data with the Processed and Pairs files

Here, we outline every step taken to merge the scraped Raw XML data with the Processed and Pairs TXT downloads from IntAct, after cleaning. Information in "intact_processed_positivePPIs.csv" was combined with the raw downloads "intact.txt" and "intact-micluster.txt". Information in "intact_processed_negativePPIs.csv" was combined with the raw downloads "intact_negative.txt" and "intact-micluster_negative.txt". In the text below, any mention of grouping or investigating "by PPI" refers to the order-agnostic set of the IntAct IDs of Interactor A and B.

1. Grouped "intact.txt" by PPI and created a mapping between each PPI and all its detected mi-scores and expansion methods.

2. Verified that any mi-score detected for a PPI in "intact(_negative).txt" matched the aggregated mi-score for that PPI in "intact-micluster(_negative).txt"
3. Utilized the mapping from part (1) to assign every row in "intact-micluster.txt" a full set of relevant expansion methods.
4. Filtered "intact-micluster(_negative).txt" to retain only rows where at least one expansion method was "not expanded". This resulted in 485,913/1,136,283 (42.7%) of positive rows and 921/931 (98.9%) of negative rows.
5. Exploded "intact-micluster(_negative).txt" along the column containing interaction IntAct IDs. The resulting database was no longer indexed by PPI and had several rows with the same PPIs and different interaction IDs (representing different studies and different experiments detecting the same PPI).
6. Merged the cleaned XML data with the appropriate "intact-micluster(_negative)" data on the following: interaction IntAct ID, PPI, interactor A IntAct ID, interactor B IntAct ID. This ensured that only one configuration of Interactor A-Interactor B was retained per PPI per interaction IntAct ID.
7. **UniProt ID Remapping**: The unique list of all UniProt IDs for Interactors A and B was collected. Rather than collecting the specific isoform provided (*e.g.,* P35240-1), we trimmed the ID down to its canonical form (*e.g.,* P35240). This list was submitted to the UniProt ID Mapping tool, under UniProt release 2025_04. Two results files were downloaded: (1) FASTA with canonical and isoform sequences, and (2) TSV with all default fields as well as Chain, Peptide, Propeptide, Signal Peptide, and Transit Peptide. These fields were added because many of IntAct's provided UniProt IDs ended with "-PRO...", indicating that they represented a chain or peptide. The chain, peptide, propeptide, signal peptide, and transit peptide sequences for each UniProt accession were extracted and added to the candidate list of sequences provided by the FASTA file. Each Interactor A and Interactor B sequence was re-matched to its correct UniProt ID with isoform/chain/peptide. In any case where the canonical isoform matched and it did not have a corresponding isoform (*e.g.,* Q10173), a stand-in isoform "-0" was appended (*e.g.,* Q10173-0), to indicate clearly that isoforms were considered for every sequence. All canonical UniProt IDs provided by IntAct were correct, unless they were deprecated (which was the case for about 2% each of interactors A and B).

These steps produced two intermediate files which represent the merging of Raw XML, Processed TXT, and Pairs TXT data. Positive interactions are stored in "merged.txt" and negative in "merged_neg.txt".

A.2.4 Incorporating mutations

The mutation annotations provided in the raw XML and "mutations.tsv" created an opportunity to extract additional positive and negative sequences. Each mutation feature is associated with an MI term, which was used to determine whether the original sequence binds its partner and whether the mutated sequence binds its partner.

According to the IntAct curation manual and Molecular Ontologies, a mutation is described as "disrupting" an interaction if and only if the interaction is completely abolished. Meanwhile,

a mutation is described as "causing" an interaction if and only if the interaction cannot occur without the mutation. Using these definitions, we assume that "decreasing" and "increasing" imply the presence of an interaction both before and after mutation (Table A2).

Table A2: Categorization of mutation-related MI terms.

MI	Definition	Original Binds	Mutated Binds
MI:2226	mutation with no effect	yes	yes
MI:0382	mutation increasing interaction	yes	yes
MI:1132	mutation increasing interaction strength	yes	yes
MI:1131	mutation increasing interaction rate	yes	yes
MI:0119	mutation decreasing interaction	yes	yes
MI:1133	mutation decreasing interaction strength	yes	yes
MI:1130	mutation decreasing interaction rate	yes	yes
MI:0573	mutation disrupting interaction	yes	no
MI:1128	mutation disrupting interaction strength	yes	no
MI:1129	mutation disrupting interaction rate	yes	no
MI:2227	mutation causing an interaction	no	yes
MI:0118	mutation	unknown	unknown
MI:2333	mutation with complex effect	unknown	unknown

The following steps were taken to incorporate mutation data into "merged.txt" (positive PPIs and PepPIs) and "merged_neg.txt" (negative PPIs and PepPIs).

1. Combined TSV and XML-derived data.

   (a) Exploded "merged" and "merged_neg" by interaction IntAct ID, so that each row contained exactly one interaction identifier.

   (b) Identified columns from XML-scraping that relate to mutations: "mutation_mi_*N*", "mutation_name_*N*", "mutation_short_*N*", "mutation_begin_*N*", "mutation_end_*N*", "mutation_orig_*N*", "mutation_new_*N*", where *N* references either interactor "A" or "B". Exploded both databases by the columns which relate to interactor A; then by the columns which relate to interactor B. This action separated mutations which were collected from the same experiment XML block, but were actually applied separately. At this point, each distinct set of mutations resides in its own row.

   (c) Separated any row where both interactor A and B had mutation-related data into two rows: one per interactor + mutation. This decision reflects an assumption that no experiment mutated both partners at the same time. (We later made an exception for homomeric interactions and assumed that in these cases, both partners were always mutated at the same time.)

   (d) Cleaned original/mutated sequence data scraped from XML such that spaces and escape sequences ("\r", "\n") were removed, *e.g.,* cleaning "QQQ\r\nQQQQ" to "QQQQQQQ".

   (e) Merged the aggregated mutation data from "mutations.tsv" with the positive and negative databases cleaned in previous steps. Merging was based on matching interaction

IntAct IDs.

(f) Dropped any rows labeled with MI:0429, "necessary binding region". Binding regions will be incorporated in a separate processing pipeline, instead of being treated as mutations.

(g) Dropped any row with no mutation data (from either XML-scraping, or the mutations TSV).

(h) In each row, determined which partner ("A" or "B") was affected by mutation. This was done by matching the "Affected protein AC" column from the mutations TSV with the interactors' database accessions, which were previously collected from XML and TXT files. The "Affected protein AC" column utilized either a UniProt, DIP, or IntAct identifier to indicate which partner was mutated.

(i) Cleaned up the formatting of starting and ending coordinates. Many coordinates were formatted like this example: "123..123-456..456". Such entries were simplified to "123-456".

(j) Filtered the current merged databases to correct, high-quality mutation annotations. To do this, we ensured that one of the following criteria was met:

   i. Mutation data was scraped from Raw XML, and not available in the Feature TSV file. (It is unclear why these cases were missed in the TSV file. We manually confirmed that several of them were correctly pulled from the raw XML.) OR,

   ii. Mutation data was available both in the Raw XML and Feature TSV file. In these cases, we were able to determine which partner was mutated (step 1h). Additionally, both the mutation range (*e.g.,* 319-319) and short label (*e.g.,* P12612:p.Arg319Ala) exactly matched between the XML-derived data and the TSV-derived data. Consistency between these critical fields confirmed that the XML-derived data had been scraped correctly.

(k) Filled in mutation data for both partners when the PPI is a homomer. As stated in step 1c, this decision reflects an assumption that for homomeric interactions, both partners are always mutated.

(l) The prior step necessitated recalculating which partners were affected by mutation. For rows with Feature TSV-derived data, we once again looked for matches with "Affected protein AC". For rows without Feature TSV-derived data, we determined if partner A, B, or both (only in homomeric interactions) were mutated.

(m) Regrouped on columns related to "Feature # AC", which do not meaningfully separate different mutation features. This fixed the problem of multiple "Feature # AC"s being assigned to mutation annotations that were otherwise the same.

(n) Dropped rows lacking amino acid sequences before and after mutation, within the specified coordinates.

(o) **Determined mutated sequences**. Utilized the Raw XML-derived "aa_*N*", "mutation_begin_*N*", "mutation_end_*N*", "mutation_orig_*N*", and "mutation_new_*N*" columns to determine

the full-length sequence of the protein after each mutation.

2. Assigned yes/no/unknown PPI labels to each pair involving a wildtype or mutated partner.

    (a) **Manually labeled mutation-related features.** In addition to MI terms (Table A2), 895 feature annotations from the "Feature annotation(s)" column and 12 feature accessions from the "# Feature AC" column were analyzed for evidence of binding in either the original or mutated sequence.

    (b) Merged the manually-labeled features with the rest of the mutation data. Feature type labels were merged on the "Feature type" column; Feature AC labels on "# Feature AC"; Feature annotations on "Feature annotation(s)".

    (c) Determined whether each wildtype/mutated sequence forms a PPI using our manual labels - on a per-row basis. At this point, we had manual yes/no/unknown labels, for each wildtype *and* mutated sequence, indicating whether that sequence binds its partner in that row. We had three separate columns containing such labels, derived from three pieces of information: MI-term (*e.g.,* MI:2226), feature annotation (*e.g.,* "comment:Mutation does not disrupt interaction"), and feature accession, which often was truly an annotation (*e.g.,* "The mutant shows no significant binding activity"). In one column, we computed the unique set of binding designations per row (*e.g.,* if MI-term = "yes", feature annotation = "yes", and feature accession = "no", the unique set would be "yes,no"). Then, in another column, we computed a final decision. If "yes" and "no" were both present, the final label would be "unknown". Otherwise, "yes" or "no" would each dominate a co-occurring label of "unknown".

    (d) **Determined whether each wildtype/mutated sequence forms a PPI using our manual labels - on a per-*sequence* basis.** In this crucial step, we aggregated evidence from different experiments pertaining to the same two sequences. We found several cases of disagreement between rows that described the same sequence pair, *e.g.,* one "yes" and one "no". In all of these cases, the disagreement could be clearly traced back to conflicting MI-terms. For example, the same mutation could be reported as "causing" and "disrupting" an interaction. These conflicts cannot be resolved, and therefore they were assigned final labels of "unknown".

3. **Created mutation-aware positive, negative, and unknown PPI databases.**

    (a) Incorporated the mutation annotations into the larger PPI database, by matching on the following fields: interaction IntAct ID, order-agnostic combination of IntAct IDs for interactors A and B, and order-agnostic combination of *wildtype* sequences for interactors A and B. Effectively, we found rows in "merged.txt" or "merged_neg.txt" that pertained to the same study and combination of interactors, and we created separate rows for the original wildtype PPI, as well as each combination of wildtype and mutated sequence with evidence.

    (b) Asserted that all sequences were valid, meaning they only contained the canonical twenty amino acids or U (selenocysteine).

    (c) Calculated one "mutation_short" label for each row. For heteromers, this label was

equal to "mutation_short_*N*" for the affected partner. For homomers, we pipe-joined the "mutation_short" labels for each interactor. For rows with no mutation annotations, the label was set equal to np.nan (effectively, None).

(d) Reorganized such that the databases were indexed on the following fields: (1) interaction IntAct ID, (2) order-agnostic combination of IntAct IDs for interactors A and B, (3) order-agnostic combination of *wildtype* sequences for interactors A and B, and (4) mutation-short label.

We note that none of the intermediate databases were sequence-indexed. We kept the databases organized by piece of evidence throughout mutation, PTM, binding site, and interaction MI-term processing. Only at the final processing step were the three databases rearranged such that each row contained a unique pair of sequences. A summary of changes in database size at various processing steps can be found in table A3.

Table A3: Quantitative summary of mutation-annotation processing. Counts in the first section refer to mutation-feature records. Counts in the second section refer to interaction-evidence rows unless otherwise indicated.

**Processing stage**	**Count**
Mutation-feature annotation processing	
Mutation-feature records retrieved	83,644
Records mappable to an interaction	83,620
Records lacking an interaction identifier	24
Records lacking an affected interactor	17
Records lacking a mutation MI term	12
Records lacking a feature range	10
Mutation-associated interaction-evidence processing	
Positive evidence rows containing at least one mutation annotation	37,655
Negative evidence rows containing at least one mutation annotation	12
After the exploding and merging steps, total rows with XML-derived or concordant mutation assignments: positive	93,177
After the exploding and merging steps, total rows with XML-derived or concordant mutation assignments: negative	14
Rows containing sufficient mutation information: positive	73,211
Rows containing sufficient mutation information: negative	13
Rows retained after joining redundant mutation-accession assignments: positive	72,771
Rows retained after reconstruction of the mutated sequence: positive	71,335
Rows retained after reconstruction of the mutated sequence: negative	13
Conflicting annotations; unique sequence pairs	
Wild-type pairs with conflicting binding outcomes	357
Mutated pairs with conflicting binding outcomes	1,153

A.2.5 Incorporating PTMs

To ensure that all amino acid sequences accurately represent the state of each binding partner, we also incorporated PTM MIs and annotations. Many PTM-related MI and MOD terms denote the type of modification (*e.g.,* psi-mi:"MI:0170"(phosphorylated residue)), which does not directly communicate binding or lack thereof. We instead utilized these terms to construct PTM-inclusive sequences.

For the categorizations below, we assume that "prerequisite-ptm" means the interaction will not occur without the PTM; "resulting-ptm" means the PTM was not present when the interaction was initiated; and terms such as "increasing" or "decreasing" imply a change in interaction strength rather than interaction gain or loss (Table A4). These assumptions are based on the IntAct curation manual definitions of MI:0925 ("observed-ptm") and its children. Additionally, several entries in the "Feature annotation(s)" column of "ptms.tsv" utilized very similar vocabulary to the MI-terms. We label both PTM-related MI terms and similar annotations in Table A4.

Table A4: Categorization of PTM-related MI-terms and feature annotations.

MI	Definition / Term	Wildtype Binds	PTM'd Binds
**Terms with PSI-MI identifiers**			
MI:0925	observed-ptm	unknown	unknown
MI:0638	prerequisite-ptm	no	yes
MI:0639	resulting-ptm	yes	unknown
MI:1233	resulting-cleavage	yes	unknown
MI:1223	ptm decreasing an interaction	yes	yes
MI:1224	ptm increasing an interaction	yes	yes
MI:1225	ptm disrupting an interaction	yes	no
**Additional annotation terms (no PSI-MI ID)**			
	phosphorylation increasing strength	yes	yes
	observed-ptm:Resulting PTM	yes	unknown
	observed-ptm:Prerequisite-PTM	no	yes

The processing steps for incorporating PTMs mirrored those taken for mutations. PTM processing also incorporated an additional quality control check, where any rows with unclear PTMs were removed. For example, if the indicated PTM region was a span of several amino acids and it was unclear if the PTM was applied to all, or if a PTM residue replaced this span in the sequence, that piece of evidence was removed from consideration. Additionally, PTMs that implied a mutation at the same time were removed, since the feature annotations do not clarify whether both a mutation and a PTM were applied (*e.g.,* original residue Serine, PTM token "MI:0165(N6-methyl-L-lysine)").

Before finalizing the PTM-aware SNOOPPI database, we checked for rows that had conflicting mutation and PTM annotations and moved these instances to unknown. Specifically, we looked for instances where the verdict on an interaction without PTMs was different whether a PTM or mutation annotation was being considered; for example, a PTM annotation may indicate that a specific pair of wild-type sequences (no PTM) interacts. However, there may be a mutation

annotation where this same pair of sequences is either the original or the mutated sequence pair, and that pair is decided not to interact. Since the labels conflict, the wild-type pair must be reassigned to unknown. Major PTM-processing steps and their consequences on dataset size are documented in Table A5. Using all PTM tokens in the final dataset, we constructed a mapping between SNOOPPI PTM tokens and PTM-Mamba [29] tokens (Tables A6, A7).

Table A5: Quantitative summary of post-translational modification (PTM) annotation processing. Feature-record counts describe the aggregated PTM annotation table. Subsequent counts describe interaction-evidence rows unless otherwise indicated. Site-level QC counts can exceed the number of evidence rows because an evidence row may contain more than one annotated PTM site.

**Processing stage**	**Count**
PTM-feature annotation processing	
PTM-feature records retrieved	10,286
Records mappable to an interaction	10,283
Records lacking an interaction identifier	3
Records lacking an affected interactor	2
Records lacking a PTM MI term	1
Records lacking a feature range	1
PTM-associated interaction-evidence processing	
Positive evidence rows containing a PTM annotation	6,455
Negative evidence rows containing a PTM annotation	4
After the exploding and merging steps, total rows containing reconciled XML-derived or aggregated PTM data: positive	13,369
After the exploding and merging steps, total rows containing reconciled XML-derived or aggregated PTM data: negative	4
Rows retained after assigning the affected partner and reconciling feature annotations: positive	6,818
Rows retained after assigning the affected partner and reconciling feature annotations: negative	4
Rows retained after joining redundant feature-accession assignments: positive	6,581
Rows retained after excluding annotations that represented mutations rather than PTMs	6,577
PTM site-level quality control	
PTM sites accepted using the original residue	6,295
PTM sites flagged for exclusion or review	589
Residue named by the PTM annotation did not match the sequence	493
Annotation represented a residue span rather than a single site	89
Reported resulting residue differed without a supported exception	7
Conflicting annotations	
Original sequence pairs with conflicting PTM-derived binding outcomes	6
PTM-containing sequence pairs with conflicting binding outcomes	12

Table A6: Mapping from SNOOPPI PTM annotation vocabulary terms to PTM-Mamba tokens. PTM-Mamba tokens without an appropriate corresponding SNOOPPI term are listed at the end.

SNOOPPI vocabulary token	Residue context	PTM-Mamba token
<MI:0160(asymmetricdimethyl-L-arginine)>	R	<Asymmetricdimethylarginine>
<MI:0170(phosphorylatedresidue)>	D	<4-aspartylphosphate>
<MI:0170(phosphorylatedresidue)>	S	<Phosphoserine>
<MI:0170(phosphorylatedresidue)>	T	<Phosphothreonine>
<MI:0170(phosphorylatedresidue)>	Y	<Phosphotyrosine>
<MI:0176(O-phospho-L-serine)>	S	<Phosphoserine>
<MI:0177(O-phospho-L-threonine)>	T	<Phosphothreonine>
<MI:0178(O4’-phospho-L-tyrosine)>	Y	<Phosphotyrosine>
<MI:0526(N-acetylatedL-lysine)>	K	<N6-acetyllysine>
<MOD:00005(O-glycosyl-L-threonine)>	T	<O-linked(GalNAc...)threonine>
<MOD:00039(4-hydroxy-L-proline)>	P	<4-hydroxyproline>
<MOD:00040(2-pyrrolidone-5-carboxylicacid(Gln))>	Q	<Pyrrolidonecarboxylicacid>
<MOD:00050(N-acetyl-L-alanine)>	A	<N-acetylalanine>
<MOD:00058(N-acetyl-L-methionine)>	M	<N-acetylmethionine>
<MOD:00064(N6-acetyl-L-lysine)>	K	<N6-acetyllysine>
<MOD:00068(N-myristoylglycine)>	G	<N-myristoylglycine>
<MOD:00078(omega-N-methyl-L-arginine)>	R	<Omega-N-methylarginine>
<MOD:00113(S-geranylgeranyl-L-cysteine)>	C	<S-geranylgeranylcysteine>
<MOD:00159(O-phosphopantetheine-L-serine)>	S	<O-(pantetheine4’-phosphoryl)serine>
<MOD:00160(N4-glycosyl-L-asparagine)>	N	<N-linked(GlcNAc...)asparagine>
<MOD:00181(O4’-sulfo-L-tyrosine)>	Y	<Sulfotyrosine>
<MOD:00394(monoacetylatedresidue)>	A	<N-acetylalanine>
<MOD:00394(monoacetylatedresidue)>	K	<N6-acetyllysine>
<MOD:00394(monoacetylatedresidue)>	M	<N-acetylmethionine>
<MOD:00394(monoacetylatedresidue)>	S	<N-acetylserine>
<MOD:00414(monomethylatedL-arginine)>	R	<Omega-N-methylarginine>
<MOD:00658(methylatedarginine)>	R	<Omega-N-methylarginine>
<MOD:00659(methylatedglutamine)>	Q	<N5-methylglutamine>
<MOD:00693(glycosylatedresidue)>	N	<N-linked(GlcNAc...)asparagine>
<MOD:00693(glycosylatedresidue)>	T	<O-linked(GalNAc...)threonine>
<MOD:00722(monomethylatedL-glutamine)>	Q	<N5-methylglutamine>
<MOD:00783(dimethylatedL-arginine)>	R	<Asymmetricdimethylarginine>
<MOD:02078(acetylatedresidue)>	A	<N-acetylalanine>
<MOD:02078(acetylatedresidue)>	K	<N6-acetyllysine>
<MOD:02078(acetylatedresidue)>	M	<N-acetylmethionine>
<MOD:02078(acetylatedresidue)>	S	<N-acetylserine>
<MI:0120(proteinmodification)>	—	—
<MI:0165(N6-methyl-L-lysine)>	K	—
<MI:0166(N6,N6-dimethyl-L-lysine)>	K	—
<MI:0167(N6,N6,N6-trimethyl-L-lysine)>	K	—
<MI:0179(uncategorizedproteinmodification)>	—	—

Continued on next page

Table A6: Mapping from source PTM annotation vocabulary terms to PTM-Mamba tokens.

SNOOPPI vocabulary token	Residue context	PTM-Mamba token
<MI:0181(L-selenomethionine)>	M	—
<MI:0189(N6-glycyl-L-lysine)>	K	—
<MI:0527(monoadenosinediphosphoribosyl(ADP-ribosyl) modifiedresidue)>	—	—
<MI:0551(nitratedresidue)>	—	—
<MI:0911(crosslinker)>	—	—
<MOD:00033(crosslinkedresidues)>	—	—
<MOD:00053(N-acetyl-L-glutamicacid)>	E	—
<MOD:00059(N-acetyl-L-proline)>	P	—
<MOD:00069(N-palmitoyl-L-cysteine)>	C	—
<MOD:00074(N-methyl-L-phenylalanine)>	F	—
<MOD:00076(symmetricdimethyl-L-arginine)>	R	—
<MOD:00086(N6-palmitoyl-L-lysine)>	K	—
<MOD:00087(N6-myristoyl-L-lysine)>	K	—
<MOD:00091(L-arginineamide)>	R	—
<MOD:00101(L-lysineamide)>	K	—
<MOD:00105(L-serineamide)>	S	—
<MOD:00111(S-farnesyl-L-cysteine)>	C	—
<MOD:00126(N6-biotinyl-L-lysine)>	K	—
<MOD:00127(N6-lipoyl-L-lysine)>	K	—
<MOD:00201(D-phenylalanine)>	F	—
<MOD:00213(chondroitinsulfateD-glucuronosyl-D-galactosyl-D-galactosyl-D-xylosyl-L-serine)>	S	—
<MOD:00215(heparansulfateD-glucuronosyl-D-galactosyl-D-galactosyl-D-xylosyl-L-serine)>	S	—
<MOD:00219(L-citrulline)>	R	—
<MOD:00256(L-methioninesulfone)>	M	—
<MOD:00267(L-cysteinesulfinicacid)>	C	—
<MOD:00304(L-leucinemethylester)>	L	—
<MOD:00332(omega-N-glucosyl-L-arginine)>	R	—
<MOD:00427(methylatedresidue)>	—	—
<MOD:00428(dihydroxylatedresidue)>	—	—
<MOD:00437(farnesylatedresidue)>	—	—
<MOD:00438(myristoylatedresidue)>	—	—
<MOD:00660(methylatedcysteine)>	C	—
<MOD:00663(methylatedlysine)>	K	—
<MOD:00674(amidatedresidue)>	—	—
<MOD:00684(deamidatedL-asparagine)>	N	—
<MOD:00685(deamidatedL-glutamine)>	Q	—
<MOD:00689(disulfidecrosslinkedresidues)>	—	—
<MOD:00699(porphyrinmodifiedresidue)>	—	—
<MOD:00714(methylatedglycine)>	G	—
<MOD:00798(halfcystine)>	C	—
<MOD:00850(unnaturalresidue)>	—	—
<MOD:00861(phosphoruscontainingmodifiedresidue)>	—	—
<MOD:00868(natural,non-standardencodedresidue)>	—	—
<MOD:00902(modifiedL-arginineresidue)>	R	—
<MOD:00905(modifiedL-cysteineresidue)>	C	—
<MOD:00908(modifiedglycineresidue)>	G	—
<MOD:00911(modifiedL-leucineresidue)>	L	—
<MOD:00912(modifiedL-lysineresidue)>	K	—

Continued on next page

Table A6: Mapping from source PTM annotation vocabulary terms to PTM-Mamba tokens.

SNOOPPI vocabulary token	Residue context	PTM-Mamba token
<MOD:00914(modifiedL-phenylalanineresidue)>	F	—
<MOD:00916(modifiedL-serineresidue)>	S	—
<MOD:00917(modifiedL-threonineresidue)>	T	—
<MOD:00919(modifiedL-tyrosineresidue)>	Y	—
<MOD:00920(modifiedL-valineresidue)>	V	—
<MOD:00985(halogenatedtyrosine)>	Y	—
<MOD:01047(monohydroxylatedlysine)>	K	—
<MOD:01110(isoprenylatedcysteine)>	C	—
<MOD:01116(S-farnesyl-L-cysteinemethylester)>	C	—
<MOD:01148(ubiquitinylatedlysine)>	K	—
<MOD:01149(sumoylatedlysine)>	K	—
<MOD:01163(guanylatedresidue)>	—	—
<MOD:01166(uridylatedresidue)>	—	—
<MOD:01186(monoacetylatedL-threonine)>	T	—
<MOD:01441(natural,standard,encodedresidue)>	—	—
<MOD:01456(N-phosphorylatedresidue)>	—	—
<MOD:01683(monomethylatedL-lysine)>	K	—
<MOD:01688(3-hydroxy-L-asparagine)>	N	—
PTM-Mamba vocabulary tokens without source vocabulary mappings		
—	—	<4-carboxyglutamate>
—	—	<N6-(pyridoxalphosphate)lysine>
—	—	<N6-carboxylysine>
—	—	<N6-succinyllysine>
—	—	<S-diacylglycerolcysteine>
—	—	<S-palmitoylcysteine>

Table A7: Summary of PTM token mapping coverage between the source PTM annotation vocabulary and PTM-Mamba vocabulary.

Vocabulary	Total tokens	Mappable tokens	Unmappable tokens	Percent mappable
SNOOPPI PTM vocabulary	97	26	71	26.8%
PTM-Mamba vocabulary	25	19	6	76.0%

A.2.6 Incorporating binding sites

Based on the definitions of binding site-related MI terms, only one term - "MI:0429: necessary binding region" - can be used to derive negative PPIs. The full description of MI:0429 in the OLS Ontology Search resource reads: "A sequence range within a molecule identified as being absolutely required for an interaction. The sequence may or may not be in direct physical contact with the interaction partner." It is a child term of the term "MI:0573: mutation disrupting interaction", whose definition reads: "Region of a molecule whose mutation or deletion totally disrupts an interaction strength or rate (in the case of interactions inferred from enzymatic reaction)." Based on these two definitions, we concluded that deletion mutants, BLOSUM62-substituted mutants, and shuffled mutants for the indicated MI:0429 region should be sequences that abolish interaction. Accordingly, we generated these sequences and added their corresponding non-PPIs to the set of SNOOPPI negatives. For the other three binding region-related terms, there is only evidence that binding *does* occur when the region is present; there is no evidence that binding ceases to occur when the region is mutated or deleted. Our categorizations of binding site-related MI terms can be found in Table A8. A quantitative summary of binding-site annotation processing is provided in Table A9. Table A10 distinguishes inferred sequence pairs from experimentally tested sequence pairs in the feature-derived portion of SNOOPPI-Negative.

Table A8: Categorization of binding site-related MI terms.

MI	Definition	Original: Binds	Removed Binding Site: Binds
MI:0429	necessary binding region	yes	no
MI:0442	sufficient binding region	yes	unknown
MI:0117	binding-associated region	yes	unknown
MI:1125	direct binding region	yes	unknown

Table A9: Quantitative summary of binding-site annotation processing and generation of binding-site-disrupted negative interactions. Feature-record counts describe the aggregated binding-region table. Subsequent counts refer to interaction-evidence rows unless explicitly identified as unique sequence pairs.

**Processing stage**	**Count**
Binding-site feature processing	
Binding-site feature records retrieved	205,660
Records mappable to an interaction	205,542
Records lacking an interaction identifier	118
Records lacking an affected interactor	84
Records lacking a binding-feature MI term	72
Records lacking a feature range	50
Binding-site-associated interaction-evidence processing	
Positive evidence rows containing a binding-site annotation	138,159
After the exploding and merging steps, total rows containing reconciled XML-derived or aggregated binding-site data	482,478
Rows retained after requiring consistency between XML-derived and aggregated annotations	185,817
Rows retained after joining redundant feature-accession assignments	171,606
Generation of disruptive sequence variants	
Partner-A rows with at least one successfully generated variant	88,356 of 88,556
Binding-region deletion generated	88,356
Binding-region shuffle generated	88,166
BLOSUM62 substitution generated	88,356
Partner-B rows with at least one successfully generated variant	86,548 of 86,738
Binding-region deletion generated	86,548
Binding-region shuffle generated	86,362
BLOSUM62 substitution generated	86,548

Table A10: Feature-derived negative PPIs in SNOOPPI.

Negative PPI category	Number of PPIs
Strictly binding-site-derived (sequences are inferred negatives)	1,824
PTM- and/or mutation-derived (experimentally tested sequences)	3,522

A.2.7 Conclusions from sequence normalization of IntAct

Each final SNOOPPI PPI has a connection to IntAct, which is encoded and searchable in the database. Sequence pairs originally present in IntAct and retained by SNOOPPI were either "confirmed" (original label matches SNOOPPI label) or "reclassified" (original label does not match SNOOPPI label). Entries outside of the MI:0915 ("physical association") tree were removed because SNOOPPI is not concerned with loose forms of association such as colocalization. Meanwhile, SNOOPPI entries newly derived from feature annotations are considered "added" interactions. Feature-determinative counts in Fig. 3A are unique modified sequence pairs (PTM, mutation, or binding-site variants) that SNOOPPI either added or reclassified relative to the exact IntAct pair. Confirmed pairs, whose IntAct and SNOOPPI labels already agreed, are excluded from the plot in order to emphasize the PPIs that would not have been present without parsing PTM, mutation, and binding-site features. MI:0915-derived PPIs refer to pairs where SNOOPPI's label was unknown, but the relaxed label, had MI:0915 been sufficient to guarantee interaction, would be either positive or negative.

Ultimately, sequence normalization substantially reduced identifier-level redundancy in the source data. While thousands of IntAct records were mapped 1:1 to a unique sequence pair, many records exhibited redundancy. In the final database, 146,653 IntAct interaction records collapse to 1,861 unique sequence pairs, with some normalized pairs corresponding to dozens of distinct interactor-ID combinations. Aggregating records at the sequence-pair level also enabled experimental evidence associated with the same molecular pair to be evaluated jointly, exposing conflicts that would remain separated in an identifier-indexed representation. At least 17,785 sequence pairs had both negative and positive evidence in the initial database and were ultimately assigned to Unknown. This normalization reduces the risk that identical molecular examples are treated as independent observations or distributed across training and test partitions in a machine learning task.

B Analysis of SNOOPPI and existing PPI databases: additional details

B.1 AUROC/AUPRC Analyses and Database Subsets

To calculate AUROC and AUPRC across STRING-Physical, SNOOPPI, TPPNI, Negatome 2.0, and SCN, subsampling was necessary. The sheer size of STRING's physical subnetwork ($>$1.1 billion pairs) rendered a full evaluation infeasible; therefore, STRING-Physical was filtered to only samples above the highest confidence threshold of 900 (42,138,136 pairs) [30]. We call this subset STRING-Physical*, signifying that the dataset contains high confidence pairs only. Due to the quadratic relationship between sequence length and inference time for Transformer models, length limits were also applied. All positive and negative datasets were filtered such that each pair of interacting/non-interacting sequences had a tokenized pair length of $\leq$ 2,000 amino acids, represented by † in the database subset name (Fig. 5A). Accounting for special tokens (BOS, EOS between sequences, and final EOS), this reduced the total sequence length to 1,997 amino acids at most. Finally, to ensure a fair evaluation between positive datasets, STRING-Physical*† was randomly subsampled to match the size of the filtered SNOOPPI-Positive†: 26,071 pairs. Identical subsets for all databases were used to evaluate each model: UPNA-PPI [8], TUnA [31], and PLM-interact [32]. We also conducted supplementary analyses where each negative PPI database was subsampled to an equal size of 4,524 sequence pairs (the size of the smallest database, SNOOPPI-Negative†).

Table B1: Dataset cardinalities for raw downloads and analysis / evaluation subsets. Non-STRING species mappability uses UniProt→species idmapping; STRING uses taxid prefixes in STRING IDs.

Family	Database	Subset	Unique PPIs	Unique partners	Partners mappable to species
Raw downloads					
STRING	STRING-Physical raw	raw download	1,182,586,133	36,676,908	
SNOOPPI	SNOOPPI-Positive raw	raw dataset	33,625	19,370	
	SNOOPPI-Negative raw	raw dataset	5,378	6,408	
	SNOOPPI-Unknown raw	raw dataset	835,083	369,390	
PPIRef	PPIRef raw	raw download	177,114	184,673	
PepPrCLIP	PepPrCLIP raw	raw download	14,150	16,597	
PPIKB	PPIKB raw	raw download	12,459	12,109	
PepMLM	PepMLM raw	raw download	16,240	18,544	
Negatome 2.0	Negatome 2.0 raw	raw download	4,972	2,774	
SCN	SCN raw	raw download	41,406	4,342	
TPPNI	TPPNI raw	raw download	2,847,123	4,881	
Cleaned subsets: used in Fig. 3-4 analyses					
STRING	STRING-Physical	20 canonical AA+U	1,114,583,998	36,335,623	36,335,623 (100.0%)
SNOOPPI	SNOOPPI-Positive	20 canonical AA+U+PTMs	33,625	19,370	18,877 (97.5%)
	SNOOPPI-Negative	20 canonical AA+U+PTMs	5,378	6,408	6,205 (96.8%)
	SNOOPPI-Unknown	20 canonical AA+U+PTMs	835,083	369,390	368,564 (99.8%)
	SNOOPPI-Pos/Neg	20 canonical AA+U+PTMs	39,003	24,004	23,399 (97.5%)
PPIRef	PPIRef	20 canonical AA+U	176,767	184,376	124,236 (67.4%)
PepPrCLIP	PepPrCLIP	20 canonical AA+U	11,895	14,119	7,710 (54.6%)
PPIKB	PPIKB	20 canonical AA+U	9,929	9,968	1,413 (14.2%)
PepMLM	PepMLM	20 canonical AA+U	7,158	9,282	6,353 (68.4%)
Negatome 2.0	Negatome 2.0	20 canonical AA+U	4,952	2,762	2,235 (80.9%)
SCN	SCN	20 canonical AA+U	41,406	4,342	2,924 (67.3%)
TPPNI	TPPNI	20 canonical AA+U	2,847,123	4,881	4,881 (100.0%)
Filtered / sampled subsets: used in Fig. 5 analyses.					
STRING	STRING-Physical*	* (confidence >=900)	42,138,136	9,543,580	9,543,580 (100.0%)
	STRING-Physical*†	*†	4,320,931	1,286,865	1,286,865 (100.0%)
	STRING-Physical*†#	*†#	26,071	51,469	51,469 (100.0%)
SNOOPPI	SNOOPPI-Positive†	†	26,071	16,519	16,519 (100.0%)
	SNOOPPI-Negative†	†	4,524	5,443	5,443 (100.0%)
Negatome 2.0	Negatome 2.0†	†	4,748	2,557	2,151 (84.1%)
	Negatome 2.0†#	†#	4,524	2,476	2,082 (84.1%)
SCN	SCN†	†	38,068	4,229	2,851 (67.4%)
	SCN†#	†#	4,524	2,596	1,338 (51.5%)
TPPNI	TPPNI†	†	2,656,960	4,763	4,763 (100.0%)
	TPPNI†#	†#	4,524	3,229	3,229 (100.0%)

† Total PPI length <= 1,997 amino acids, and partners have canonical 20 AAs only.
\# Size-matched, random subsample

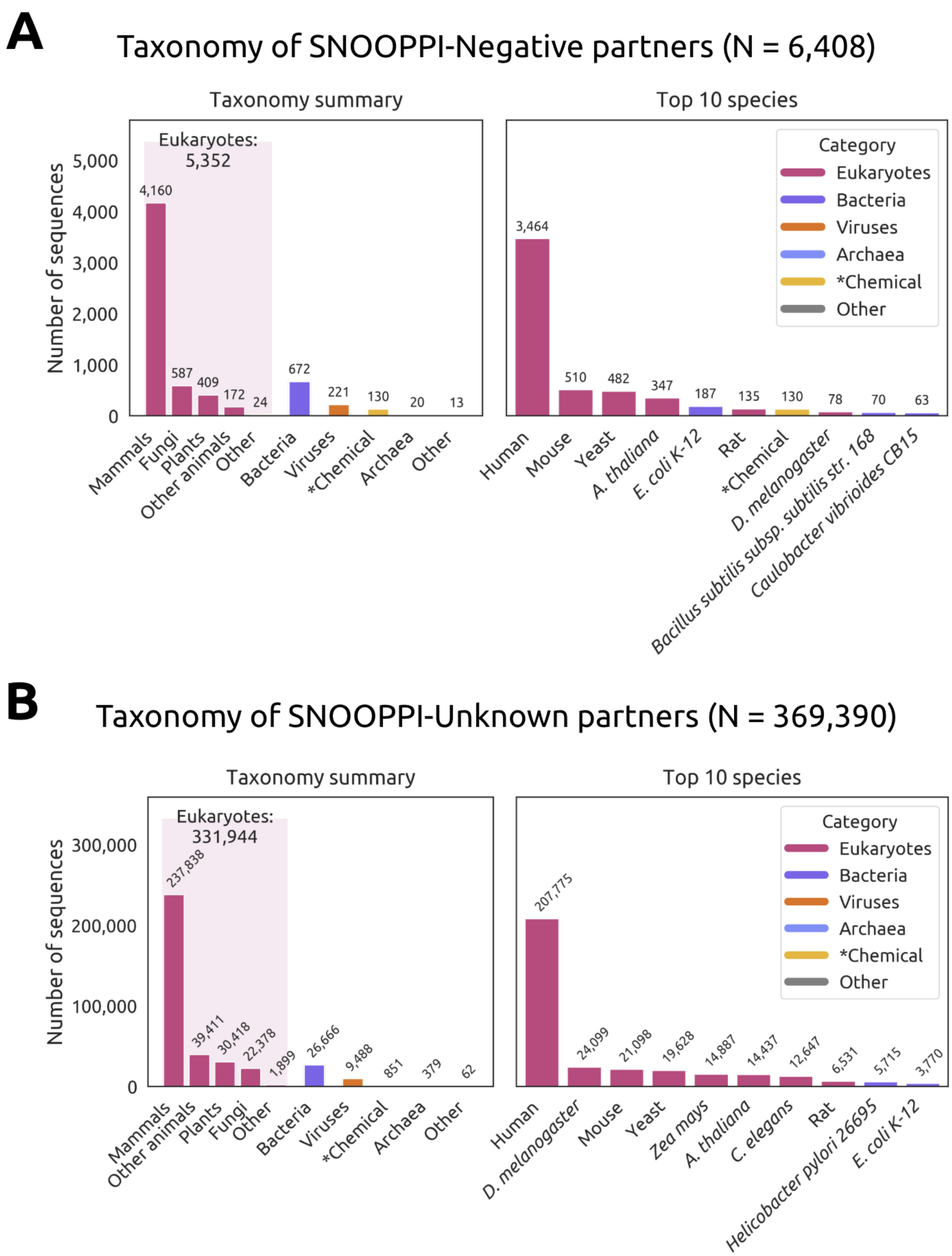

Figure B1: **Taxonomic distribution of SNOOPPI-Negative and SNOOPPI-Unknown PPI partners.** Higher-order taxonomic groups (left) and top ten species/sources for **A** SNOOPPI-Negative and **B** SNOOPPI-Unknown. *Chemical represents chemical synthesis, typically referring to a peptide.

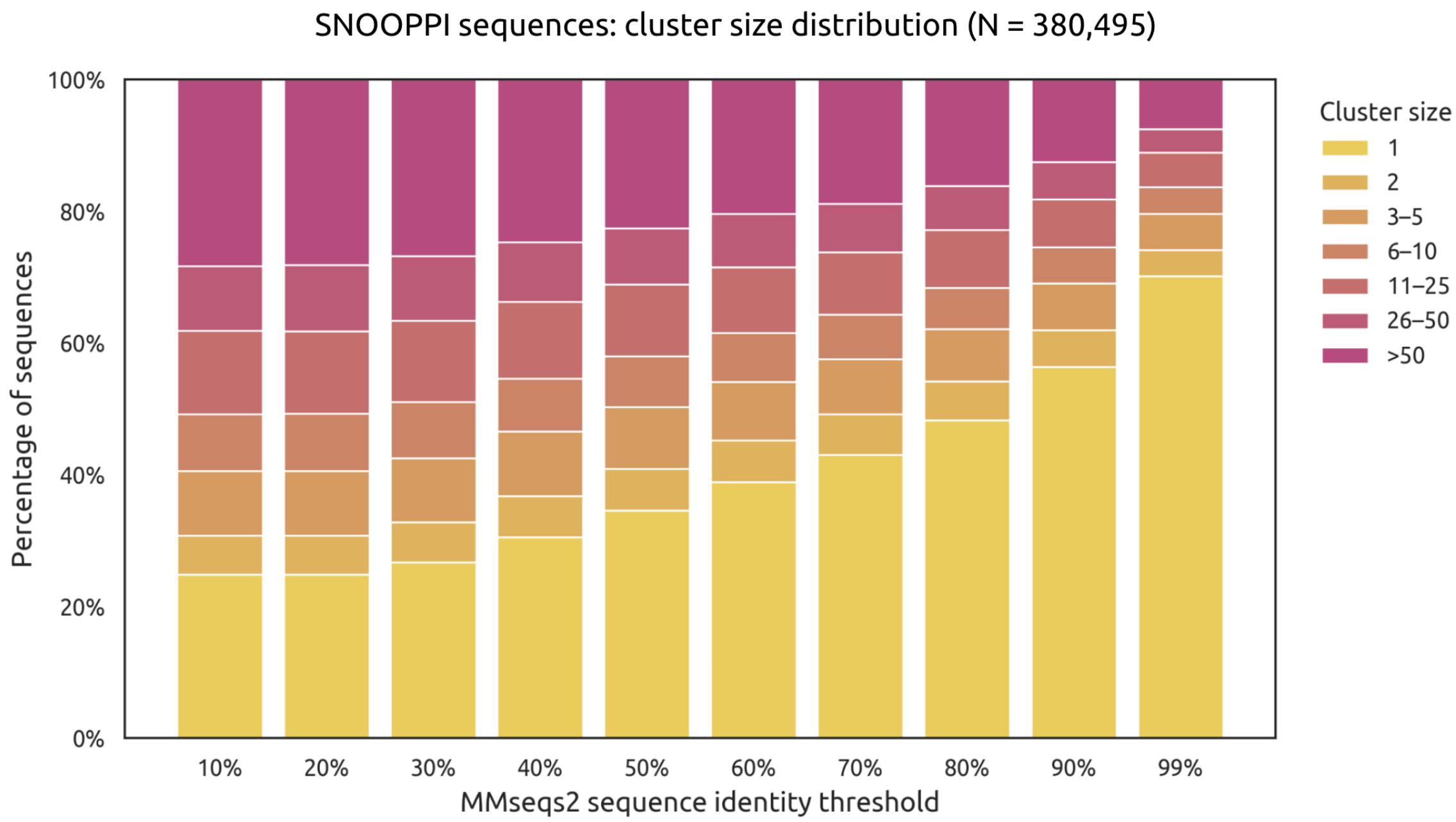

Figure B2: **Cluster size distribution at various MMseqs2 clustering percent identities, 10% through 99%.** Includes PPI partners in SNOOPPI-Positive, SNOOPPI-Negative, and SNOOPPI-Unknown.

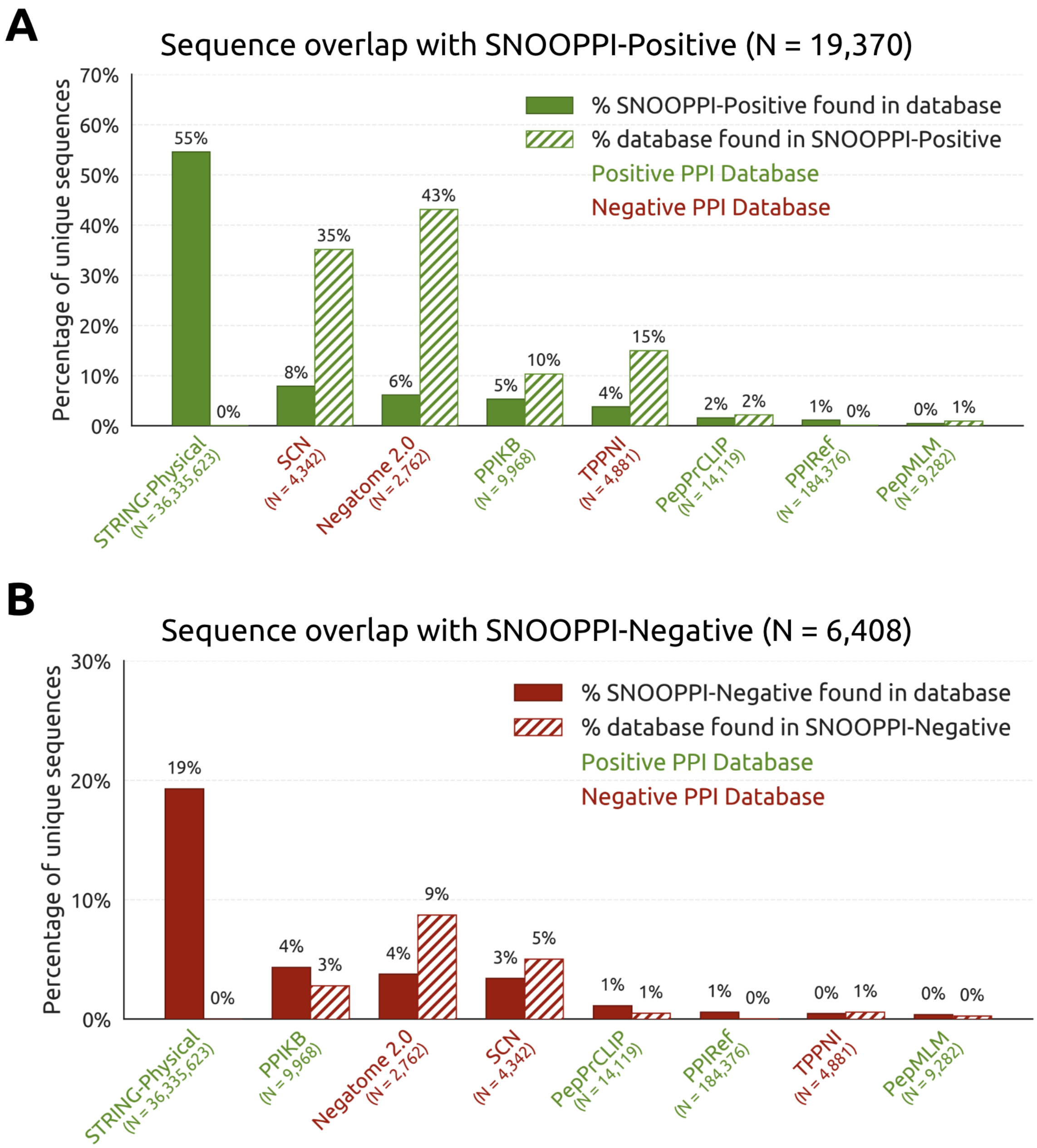

Figure B3: **Reciprocal sequence overlap between SNOOPPI subsets and comparator databases.** Overlap between STRING, SCN, Negatome 2.0, PPIKB, TPPNI, PepPrCLIP, PPIRef, and PepMLM with **A** SNOOPPI-Positive and **B** SNOOPPI-Negative. Databases are sorted by % SNOOPPI found in database, in descending order. Positive PPI databases are indicated in green font, and negative PPI databases in red. Total number of unique PPI/non-PPI partner sequences (N) is indicated for each database.

Table B2: Reciprocal unique-sequence overlap between SNOOPPI reference sets and comparison databases. N_{ref} indicates size of the SNOOPPI database; N_{db} indicates size of the comparator database; N_{shared} gives overlap between the two.

Database	N_{ref}	N_{db}	N_{shared}	% ref covered	% db in ref
SNOOPPI-Pos/Neg					
STRING-Physical	24,004	36,335,623	10,573	44.0	0.029
SCN		4,342	1,526	6.4	35.1
Negatome 2.0		2,762	1,192	5.0	43.2
PPIKB		9,968	1,032	4.3	10.4
TPPNI		4,881	731	3.0	15.0
PepPrCLIP		14,119	315	1.3	2.2
PPIRef		184,376	228	0.95	0.12
PepMLM		9,282	91	0.38	0.98
SNOOPPI-Positive					
STRING-Physical	19,370	36,335,623	10,565	54.5	0.029
SCN		4,342	1,526	7.9	35.1
Negatome 2.0		2,762	1,191	6.1	43.1
PPIKB		9,968	1,030	5.3	10.3
TPPNI		4,881	731	3.8	15.0
PepPrCLIP		14,119	308	1.6	2.2
PPIRef		184,376	222	1.1	0.12
PepMLM		9,282	89	0.46	0.96
SNOOPPI-Negative					
STRING-Physical	6,408	36,335,623	1,235	19.3	0.003
PPIKB		9,968	278	4.3	2.8
Negatome 2.0		2,762	241	3.8	8.7
SCN		4,342	218	3.4	5.0
PepPrCLIP		14,119	72	1.1	0.51
PPIRef		184,376	38	0.59	0.021
TPPNI		4,881	29	0.45	0.59
PepMLM		9,282	24	0.37	0.26

Sections use different SNOOPPI reference sets (Pos/Neg union, Positive only, Negative only).

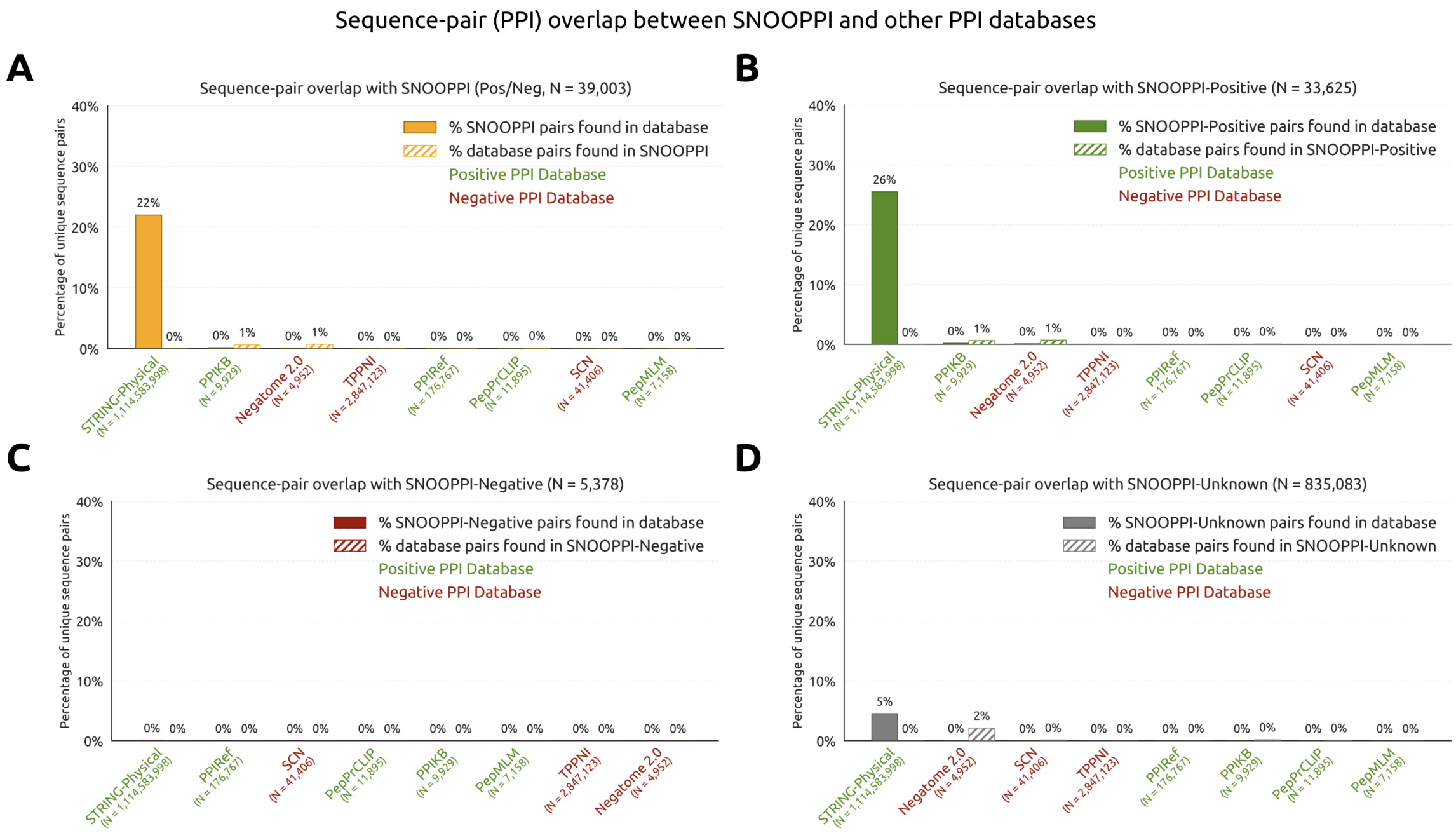

Figure B4: **Reciprocal sequence pair overlap between SNOOPPI subsets and comparator databases.** Overlap between STRING, SCN, Negatome 2.0, PPIKB, TPPNI, PepPrCLIP, PPIRef, and PepMLM with **A** SNOOPPI-Positive + SNOOPPI-Negative, **B** SNOOPPI-Positive, **C** SNOOPPI-Negative, **D** SNOOPPI-Unknown. Databases are sorted by % SNOOPPI pairs found in database, in descending order. Positive PPI databases are indicated in green font, and negative PPI databases in red. Total number of unique sequence pairs (N) is indicated for each database.

Table B3: Reciprocal unique-pair overlap between SNOOPPI reference sets and comparison databases. N_{ref} indicates size of the SNOOPPI database; N_{db} indicates size of the comparator database; N_{shared} gives overlap between the two.

Database	N_{ref}	N_{db}	N_{shared}	% ref covered	% db in ref
SNOOPPI-Pos/Neg					
STRING-Physical	39,003	1,114,583,998	8,580	22.0	0.001
PPIKB		9,929	64	0.16	0.64
Negatome 2.0		4,952	35	0.09	0.71
TPPNI		2,847,123	17	0.044	0.001
PPIRef		176,767	14	0.036	0.008
PepPrCLIP		11,895	12	0.031	0.10
SCN		41,406	3	0.008	0.007
PepMLM		7,158	0	0.0	0.0
SNOOPPI-Positive					
STRING-Physical	33,625	1,114,583,998	8,576	25.5	0.001
PPIKB		9,929	64	0.19	0.64
Negatome 2.0		4,952	35	0.10	0.71
TPPNI		2,847,123	17	0.05	0.001
PPIRef		176,767	14	0.042	0.008
PepPrCLIP		11,895	12	0.036	0.10
SCN		41,406	3	0.009	0.007
PepMLM		7,158	0	0.0	0.0
SNOOPPI-Negative					
STRING-Physical	5,378	1,114,583,998	4	0.07	0.000
PPIRef		176,767	0	0.0	0.0
SCN		41,406	0	0.0	0.0
PepPrCLIP		11,895	0	0.0	0.0
PPIKB		9,929	0	0.0	0.0
PepMLM		7,158	0	0.0	0.0
TPPNI		2,847,123	0	0.0	0.0
Negatome 2.0		4,952	0	0.0	0.0
SNOOPPI-Unknown					
STRING-Physical	835,083	1,114,583,998	37,827	4.5	0.003
Negatome 2.0		4,952	105	0.013	2.1
SCN		41,406	49	0.006	0.12
TPPNI		2,847,123	41	0.005	0.001
PPIRef		176,767	31	0.004	0.018
PPIKB		9,929	18	0.002	0.18
PepPrCLIP		11,895	7	0.001	0.06
PepMLM		7,158	0	0.0	0.0

Sections use different SNOOPPI reference pair sets (Pos/Neg union, Positive, Negative, Unknown).

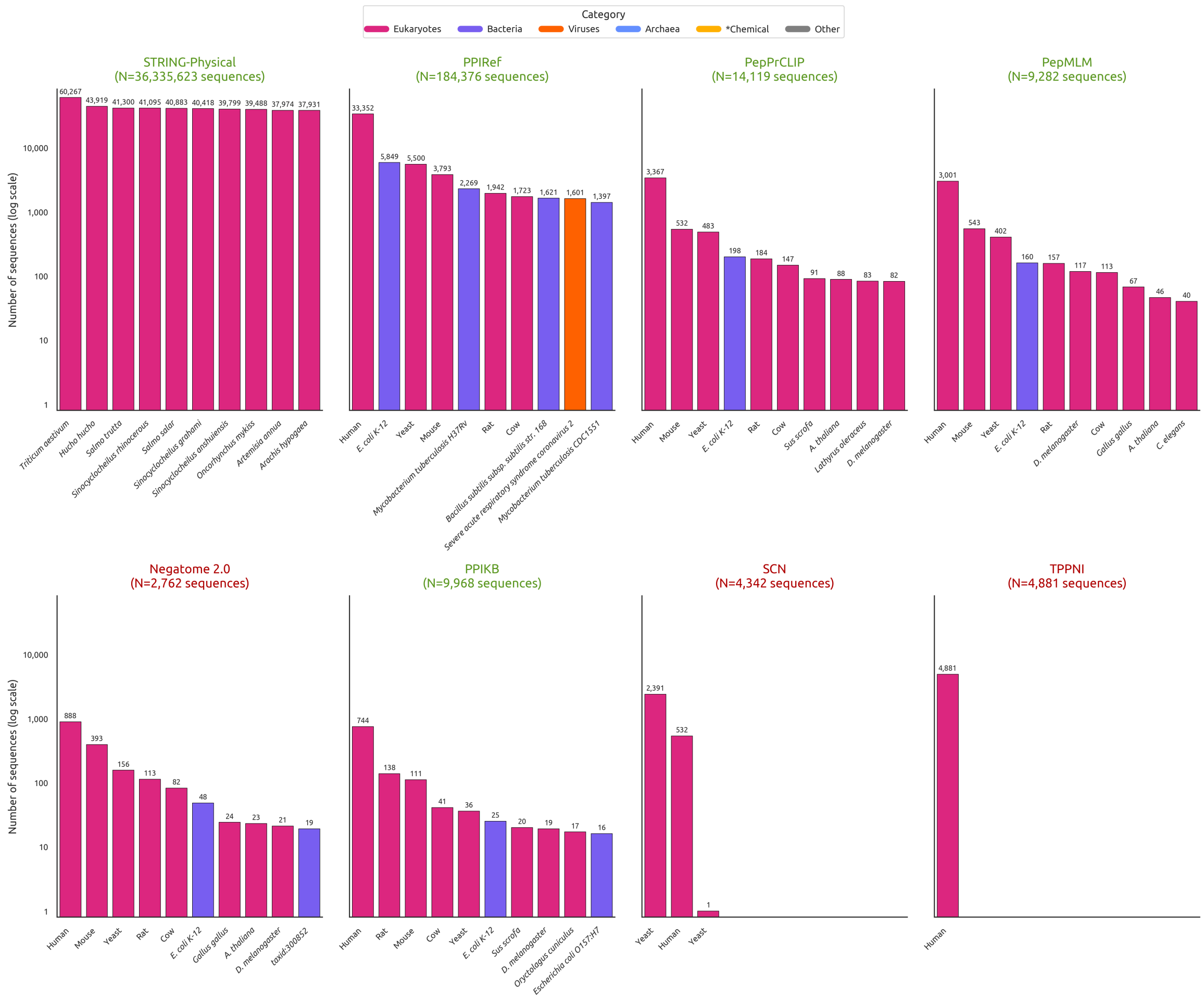

Figure B5: **Top 10 species per PPI database.** For STRING-Physical, PPIRef, PepPrCLIP, PepMLM, Negatome 2.0, PPIKB, SCN, and TPPNI, the top 10 species, by number of unique PPI partners present in the database, are displayed in descending order with a log scale. Positive PPI databases are displayed in green font, and negative PPI databases in red. Total number of unique PPI/non-PPI partner sequences in each database (N) is included.

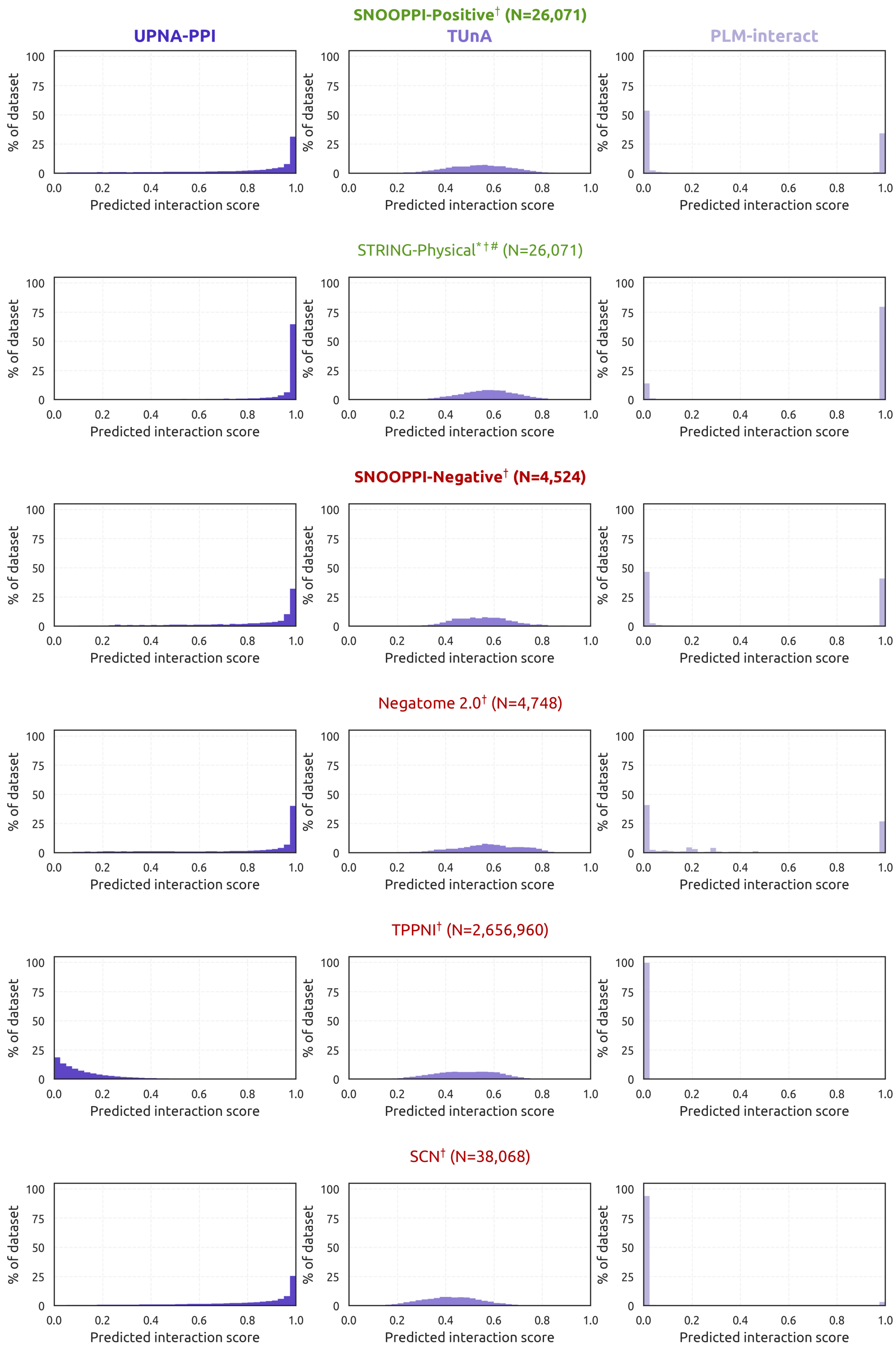

Figure B6: **Distribution of PPI classifier outputs.** UPNA-PPI, TUnA, and PLM-interact raw score distributions for each positive and negative PPI database used in the classifier analysis: SNOOPPI-Positive†, STRING-Physical*†#, SNOOPPI-Negative†, Negatome 2.0†, TPPNI†, and SCN†. Font color indicates positive (green) or negative (red) PPI database. Total number of PPI pairs in database (N) indicated.

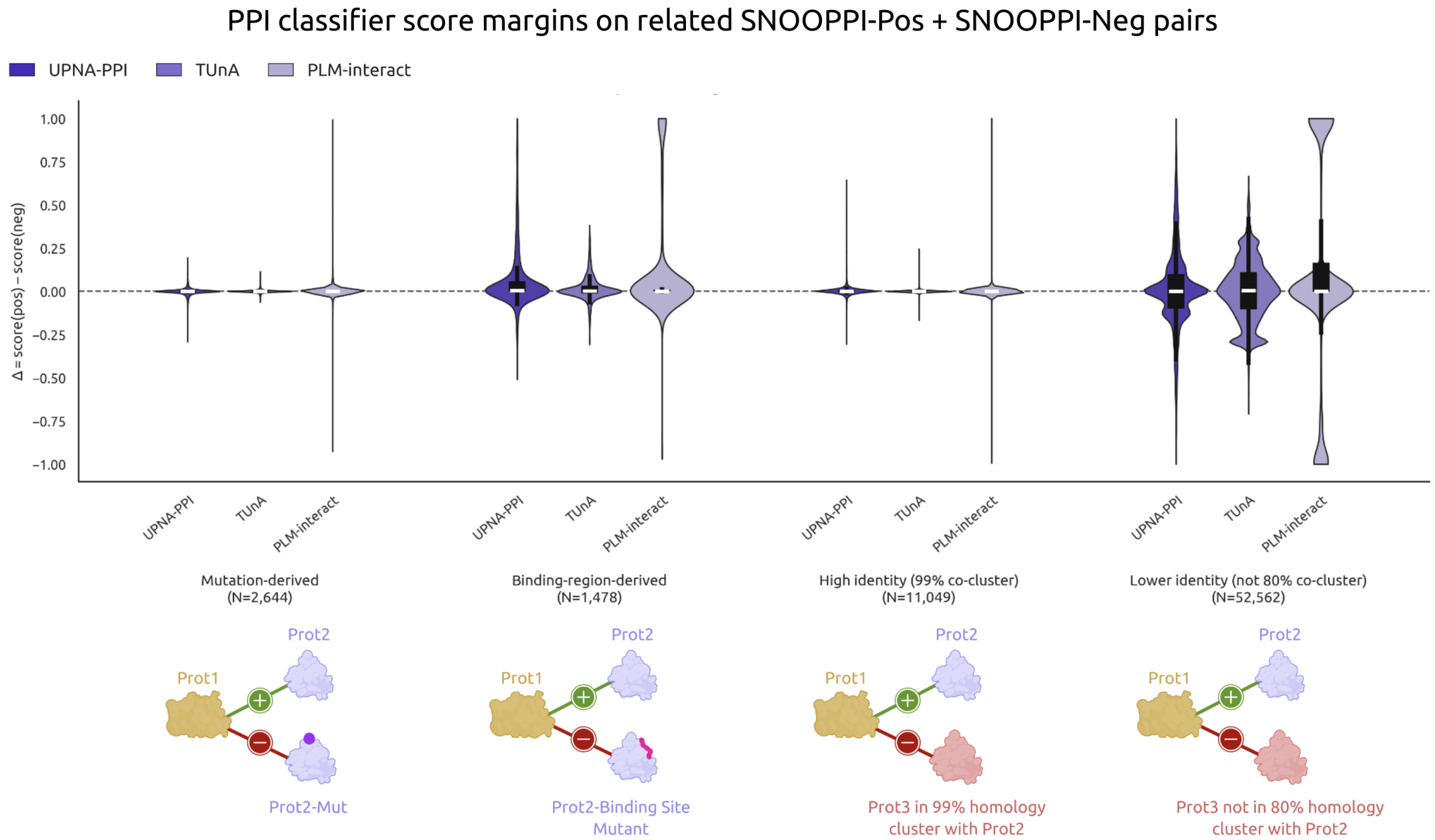

Figure B7: **PPI classifier score margins on SNOOPPI-Positive and SNOOPPI-Negative pairs sharing a biological relationship.** $\Delta = \text{score}(\text{pos}) - \text{score}(\text{neg})$, where pos and neg share one anchor protein and differ in the second partner. In mutation-derived samples (leftmost), the positive and negative partners are single- or few-amino-acid mutants of the same sequence. In binding-region-derived samples (second leftmost), the positive and negative partners differ by a SNOOPPI binding site mutation (either deletion, BLOSUM62 substitution, or shuffle). In high identity samples (second rightmost), the negative PPI partner lies in the same 99% homology cluster as the positive PPI partner. In lower identity samples (rightmost), the negative PPI partner does *not* lie in the 80% homology cluster of the positive PPI partner, indicating <80% sequence homology. Total number of samples (positive PPI + negative PPI) per category is indicated by N. Created in BioRender. Chatterjee, P. (2026) https://BioRender.com/u7pazpr

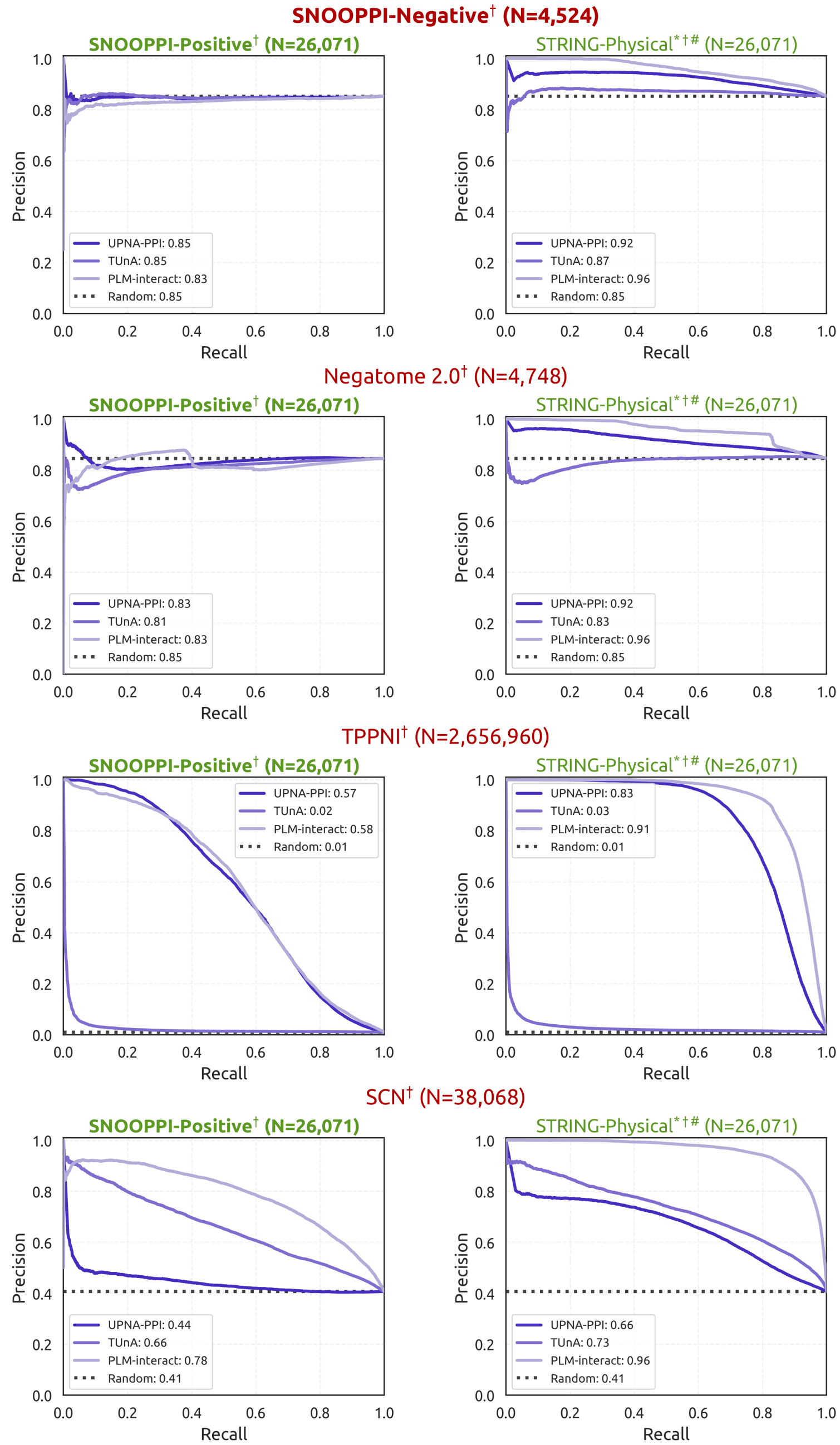

Figure B8: **PPI classifier AUPRC curves on positive and negative PPI databases.** AUPRC shown for each pairing of positive (SNOOPPI-Positive†, STRING-Physical*†#) and negative (SNOOPPI-Negative†, Negatome 2.0†, TPPNI†, SCN†) PPI databases. Random baseline is equal to the fraction of positive PPIs.

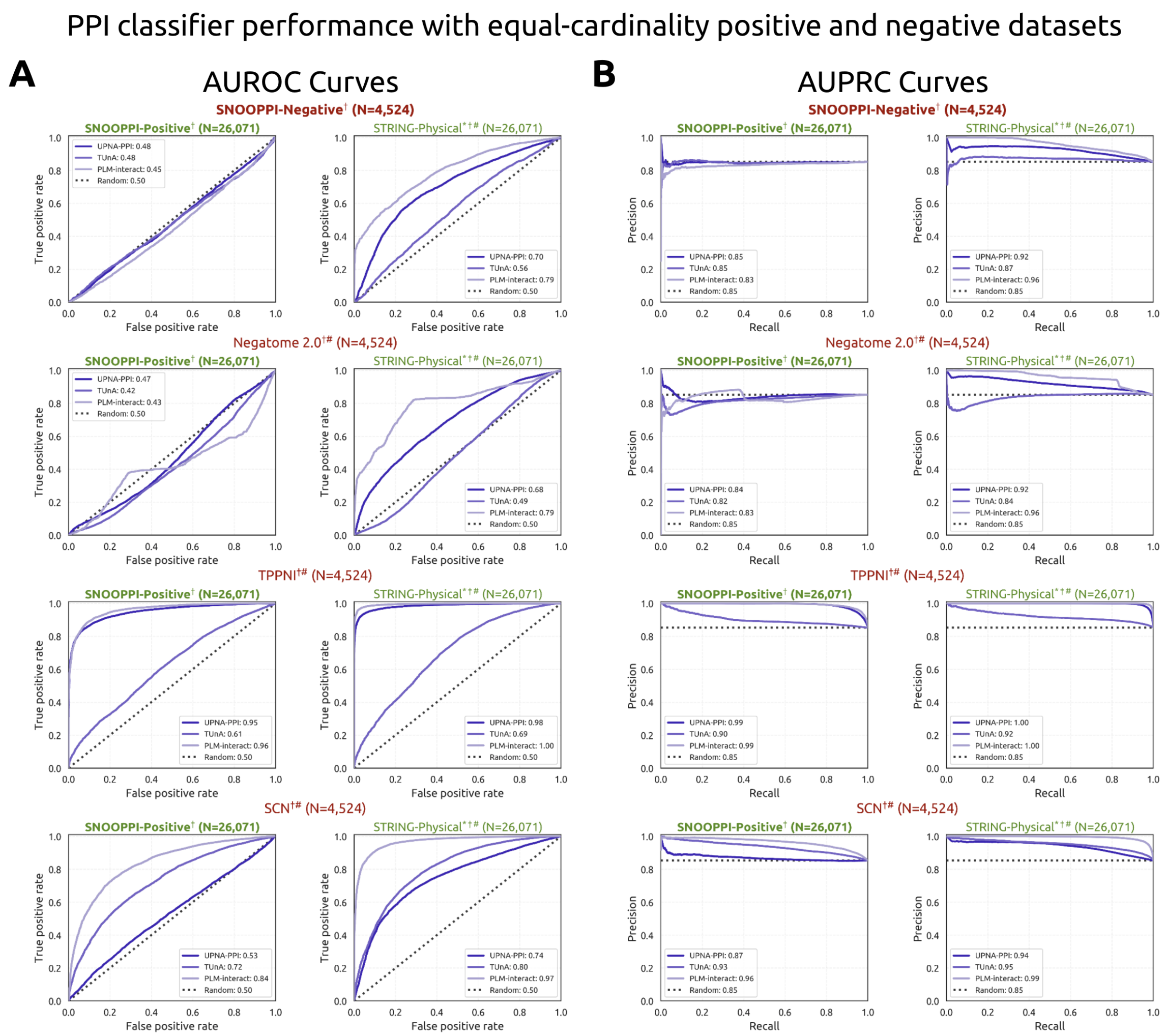

Figure B9: **PPI classifier curves with equal cardinality among all positive PPI databases (N = 26,071) and among all negative PPI databases (N = 4,524).** Negative databases were subsampled to match the smallest cardinality (SNOOPPI-Negative†). **A** AUROC and **B** AUPRC for each pair of negative (SNOOPPI-Negative†, Negatome 2.0†#, TPPNI†#, SCN†#) and positive (SNOOPPI-Positive†, STRING-Physical*†#) databases.

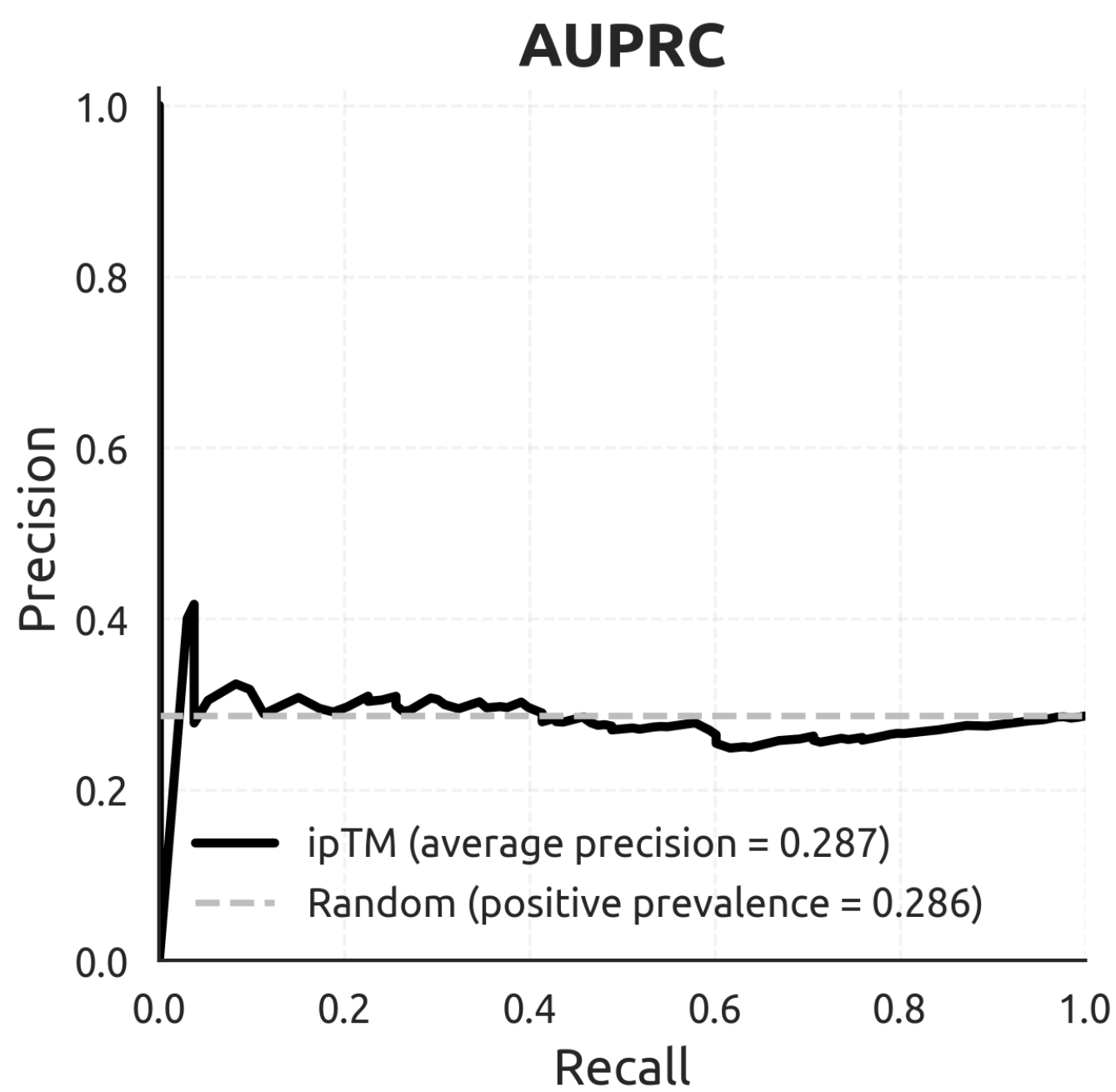

Figure B10: **AlphaFold-Multimer AUPRC on SNOOPPI-Positive and SNOOPPI-Negative mutation pairs.** Random baseline is equal to the fraction of positive PPIs.

Supplementary References

[1] Anton Bushuiev, Roman Bushuiev, Petr Kouba, Anatolii Filkin, Marketa Gabrielova, Michal Gabriel, Jiri Sedlar, Tomas Pluskal, Jiri Damborsky, Stanislav Mazurenko, and Josef Sivic. Learning to design protein-protein interactions with enhanced generalization. In *The Twelfth International Conference on Learning Representations*, 2024.

[2] Ning Zhu, Yanyu Ming, Chengyun Zhang, Sen Cao, Chongyang Li, Yaling Wu, Jingjing Guo, and Hongliang Duan. A data resource of peptide–protein interaction structures and binding affinities for peptide drug discovery. *Scientific Data*, 2026. ISSN 2052-4463. doi: 10.1038/s41597-026-07962-1. URL http://dx.doi.org/10.1038/s41597-026-07962-1.

[3] Suhaas Bhat, Kalyan Palepu, Lauren Hong, Joey Mao, Tianzheng Ye, Rema Iyer, Lin Zhao, Tianlai Chen, Sophia Vincoff, Rio Watson, Tian Z. Wang, Divya Srijay, Venkata Srikar Kavirayuni, Kseniia Kholina, Shrey Goel, Pranay Vure, Aniruddha J. Deshpande, Scott H. Soderling, Matthew P. DeLisa, and Pranam Chatterjee. De novo design of peptide binders to conformationally diverse targets with contrastive language modeling. *Science Advances*, 11(4): eadr8638, 2025.

[4] Leo Tianlai Chen, Zachary Quinn, Madeleine Dumas, Christina Peng, Lauren Hong, Moises Lopez-Gonzalez, Alexander Mestre, Rio Watson, Sophia Vincoff, Lin Zhao, Jianli Wu, Audrey Stavrand, Mayumi Schaepers-Cheu, Tian Zi Wang, Divya Srijay, Connor Monticello, Pranay Vure, Rishab Pulugurta, Sarah Pertsemlidis, Kseniia Kholina, Shrey Goel, Matthew P. DeLisa, Jen-Tsan Ashley Chi, Ray Truant, Hector C. Aguilar, and Pranam Chatterjee. Target sequence-conditioned design of peptide binders using masked language modeling. *Nature Biotechnology*, pages 1–9, 2025.

[5] Damian Szklarczyk, Rebecca Kirsch, Mikaela Koutrouli, Katerina Nastou, Farrokh Mehryary, Radja Hachilif, Annika L Gable, Tao Fang, Nadezhda T Doncheva, Sampo Pyysalo, Peer Bork, Lars J Jensen, and Christian von Mering. The STRING database in 2023: protein–protein association networks and functional enrichment analyses for any sequenced genome of interest. *Nucleic Acids Research*, 51(D1):D638–D646, 2023.

[6] Muhao Chen, Chelsea J-T Ju, Guangyu Zhou, Xuelu Chen, Tianran Zhang, Kai-Wei Chang, Carlo Zaniolo, and Wei Wang. Multifaceted protein–protein interaction prediction based on Siamese residual RCNN. *Bioinformatics*, 35(14):i305–i314, 2019.

[7] Philipp Blohm, Goar Frishman, Pawel Smialowski, Florian Goebels, Benedikt Wachinger, Andreas Ruepp, and Dmitrij Frishman. Negatome 2.0: a database of non-interacting proteins derived by literature mining, manual annotation and protein structure analysis. *Nucleic Acids Research*, 42(D1):D396–D400, 2014.

[8] Ayan Chatterjee, Babak Ravandi, Parham Haddadi, Naomi H Philip, Mario Abdelmessih, William R Mowrey, Piero Ricchiuto, Yupu Liang, Wei Ding, Juan Carlos Mobarec, and Tina Eliassi-Rad. Topology-driven negative sampling enhances generalizability in protein–protein interaction prediction. *Bioinformatics*, 41(5):btaf148, 2025.

[9] Helen M Berman, John Westbrook, Zukang Feng, Gary Gilliland, Talapady N Bhat, Helge

Weissig, Ilya N Shindyalov, and Philip E Bourne. The Protein Data Bank. *Nucleic Acids Research*, 28(1):235–242, 2000.

[10] Osama Abdin, Satra Nim, Han Wen, and Philip M Kim. PepNN: a deep attention model for the identification of peptide binding sites. *Communications biology*, 5(1):503, 2022.

[11] Pedro Martins, Diego Mariano, Frederico Chaves Carvalho, Luana Luiza Bastos, Lucas Moraes, Vivian Paixão, and Raquel Cardoso de Melo-Minardi. Propedia v2.3: A novel representation approach for the peptide-protein interaction database using graph-based structural signatures. *Frontiers in Bioinformatics*, 3:1103103, 2023.

[12] Martin Steinegger and Johannes Söding. MMseqs2 enables sensitive protein sequence searching for the analysis of massive data sets. *Nature Biotechnology*, 35(11):1026–1028, 2017.

[13] Yanzhi Guo, Lezheng Yu, Zhining Wen, and Menglong Li. Using support vector machine combined with auto covariance to predict protein–protein interactions from protein sequences. *Nucleic Acids Research*, 36(9):3025–3030, 2008.

[14] Xiao-Yong Pan, Ya-Nan Zhang, and Hong-Bin Shen. Large-scale prediction of human protein-protein interactions from amino acid sequence based on latent topic features. *Journal of Proteome Research*, 9(10):4992–5001, 2010.

[15] Pawel Smialowski, Philipp Pagel, Philip Wong, Barbara Brauner, Irmtraud Dunger, Gisela Fobo, Goar Frishman, Corinna Montrone, Thomas Rattei, Dmitrij Frishman, and Andreas Ruepp. The Negatome database: a reference set of non-interacting protein pairs. *Nucleic Acids Research*, 38:D540–D544, November 2009. ISSN 1362-4962.

[16] Noemi del Toro, Anjali Shrivastava, Eliot Ragueneau, Birgit Meldal, Colin Combe, Elisabet Barrera, Livia Perfetto, Karyn How, Prashansa Ratan, Gautam Shirodkar, Odilia Lu, Bálint Mészáros, Xavier Watkins, Sangya Pundir, Luana Licata, Marta Iannuccelli, Matteo Pellegrini, Maria Jesus Martin, Simona Panni, Margaret Duesbury, Sylvain D Vallet, Juri Rappsilber, Sylvie Ricard-Blum, Gianni Cesareni, Lukasz Salwinski, Sandra Orchard, Pablo Porras, Kalpana Panneerselvam, and Henning Hermjakob. The IntAct database: efficient access to fine-grained molecular interaction data. *Nucleic Acids Research*, 50(D1):D648–D653, 2022.

[17] Ioannis Xenarios, Lukasz Salwinski, Xiaoqun Joyce Duan, Patrick Higney, Sul-Min Kim, and David Eisenberg. DIP, the database of interacting proteins: a research tool for studying cellular networks of protein interactions. *Nucleic Acids Research*, 30(1):303–305, 2002.

[18] Andreas Zanzoni, Luisa Montecchi-Palazzi, Michele Quondam, Gabriele Ausiello, Manuela Helmer-Citterich, and Gianni Cesareni. MINT: a molecular interaction database. *FEBS letters*, 513(1):135–140, 2002.

[19] Kasun W Samarasinghe, Max Kotlyar, Sylvain D Vallet, Catherine Hayes, Alexandra Naba, Igor Jurisica, Frédérique Lisacek, and Sylvie Ricard-Blum. MatrixDB 2024: an increased coverage of extracellular matrix interactions, a new network explorer and a new web interface. *Nucleic Acids Research*, 53(D1):D1677–D1682, 2025.

[20] Max Kotlyar, Chiara Pastrello, Mark Abovsky, Alexandru Mizeranschi, Armand Keating, Luiz-

Claudio Cameron, Vinod Chandran, and Igor Jurisica. IID 2025: Physical protein interaction data with detection types, co-purified protein sets, molecular docking, and immune cell networks. *Nucleic Acids Research*, page gkaf1259, 2025.

[21] Karin Breuer, Amir K Foroushani, Matthew R Laird, Carol Chen, Anastasia Sribnaia, Raymond Lo, Geoffrey L Winsor, Robert EW Hancock, Fiona SL Brinkman, and David J Lynn. InnateDB: systems biology of innate immunity and beyond—recent updates and continuing curation. *Nucleic Acids Research*, 41(D1):D1228–D1233, 2013.

[22] The UniProt Consortium. UniProt: the universal protein knowledgebase in 2025. *Nucleic Acids Research*, 53(D1):D609–D617, 2025.

[23] Matthew Thakur, Catherine Brooksbank, Robert D Finn, Helen V Firth, Julia Foreman, Mallory Freeberg, Kim T Gurwitz, Melissa Harrison, David Hulcoop, Sarah E Hunt, Andrew R. Leach, Mariia Levchenko, Diana Marques, Ellen M McDonagh, Aziz Mithani, Helen Parkinson, Yasset Perez-Riverol, Zinaida Perova, Ugis Sarkans, Santosh Tirunagari, Eleni Tzampatzopoulou, Aravind Venkatesan, Juan-Antonio Vizcaino, Benjamin Wingfield, Barbara Zdrazil, and Johanna McEntyre. EMBL's european bioinformatics institute (EMBL-EBI) in 2024. *Nucleic Acids Research*, 53(D1):D10–D19, 11 2024. ISSN 1362-4962.

[24] Ulrich Güldener, Martin Münsterkötter, Matthias Oesterheld, Philipp Pagel, Andreas Ruepp, Hans-Werner Mewes, and Volker Stümpflen. MPact: the MIPS protein interaction resource on yeast. *Nucleic Acids Research*, 34:D436–D441, 2006.

[25] Gary D Bader, Doron Betel, and Christopher WV Hogue. BIND: the biomolecular interaction network database. *Nucleic Acids Research*, 31(1):248–250, 2003.

[26] Johannes Goll, Seesandra V Rajagopala, Shen C Shiau, Hank Wu, Brian T Lamb, and Peter Uetz. MPIDB: the microbial protein interaction database. *Bioinformatics*, 24(15):1743–1744, 2008.

[27] Mais G Ammari, Cathy R Gresham, Fiona M McCarthy, and Bindu Nanduri. HPIDB 2.0: a curated database for host–pathogen interactions. *Database*, 2016:baw103, 2016.

[28] M. Sivade, D. Alonso-López, M. Ammari, G. Bradley, N. H. Campbell, A. Ceol, G. Cesareni, C. Combe, J. De Las Rivas, N. del Toro, J. Heimbach, H. Hermjakob, I. Jurisica, M. Koch, L. Licata, R. C. Lovering, D. J. Lynn, B. H. M. Meldal, G. Micklem, S. Panni, P. Porras, S. Ricard-Blum, B. Roechert, L. Salwinski, A. Shrivastava, J. Sullivan, N. Thierry-Mieg, Y. Yehudi, K. Van Roey, and S. Orchard. Encompassing new use cases-level 3.0 of the HUPO-PSI format for molecular interactions. *BMC bioinformatics*, 19(1):134, 2018.

[29] Fred Zhangzhi Peng, Chentong Wang, Tong Chen, Benjamin Schussheim, Sophia Vincoff, and Pranam Chatterjee. PTM-Mamba: a PTM-aware protein language model with bidirectional gated Mamba blocks. *Nature Methods*, 22(5):945–949, April 2025. ISSN 1548-7105.

[30] Damian Szklarczyk, Katerina Nastou, Mikaela Koutrouli, Rebecca Kirsch, Farrokh Mehryary, Radja Hachilif, Dewei Hu, Matteo E Peluso, Qingyao Huang, Tao Fang, Nadezhda T Doncheva, Sampo Pyysalo, Peer Bork, Lars J Jensen, and Christian von Mering. The STRING database in 2025: protein networks with directionality of regulation. *Nucleic Acids Research*, 53(D1): D730–D737, 2025.

[31] Young Su Ko, Jonathan Parkinson, Cong Liu, and Wei Wang. TUnA: an uncertainty-aware transformer model for sequence-based protein–protein interaction prediction. *Briefings in Bioinformatics*, 25(5), 2024.

[32] Dan Liu, Francesca Young, Kieran D Lamb, Adalberto Claudio Quiros, Alexandrina Pancheva, Crispin J Miller, Craig Macdonald, David L Robertson, and Ke Yuan. Plm-interact: extending protein language models to predict protein-protein interactions. *Nature Communications*, 16 (1):9012, 2025.
